# Processing of visually evoked innate fear by a non-canonical thalamic pathway

**DOI:** 10.1038/ncomms7756

**Published:** 2015-04-09

**Authors:** Pengfei Wei, Nan Liu, Zhijian Zhang, Xuemei Liu, Yongqiang Tang, Xiaobin He, Bifeng Wu, Zheng Zhou, Yaohan Liu, Juan Li, Yi Zhang, Xuanyi Zhou, Lin Xu, Lin Chen, Guoqiang Bi, Xintian Hu, Fuqiang Xu, Liping Wang

**Affiliations:** 1 Shenzhen Key Lab of Neuropsychiatric Modulation and Collaborative Innovation Center for Brain Science, CAS Center for Excellence in Brain Science, Shenzhen Institutes of Advanced Technology, Chinese Academy of Sciences, Shenzhen 518055, China; 2 Wuhan Institute of Physics and Mathematics, CAS Center for Excellence in Brain Science, Chinese Academy of Sciences, Wuhan 430071, China; 3 College of Life Science and Technology, Huazhong University of Science and Technology, Wuhan 430071, China; 4 College of Life Science, Wuhan University, Wuhan 430071, China; 5 Institute of Zoology, CAS Center for Excellence in Brain Science, Chinese Academy of Sciences, Kunming 650223, China; 6 State Key Laboratory of Brain and Cognitive Science, Institute of Biophysics, CAS Center for Excellence in Brain Science, Chinese Academy of Sciences, Beijing 100101, China; 7 CAS Key Laboratory of Brain Function and Disease, and School of Life Sciences, CAS Center for Excellence in Brain Science, The University of Science and Technology of China, Hefei 230026, China

## Abstract

The ability of animals to respond to life-threatening stimuli is essential for survival. Although vision provides one of the major sensory inputs for detecting threats across animal species, the circuitry underlying defensive responses to visual stimuli remains poorly defined. Here, we investigate the circuitry underlying innate defensive behaviours elicited by predator-like visual stimuli in mice. Our results demonstrate that neurons in the superior colliculus (SC) are essential for a variety of acute and persistent defensive responses to overhead looming stimuli. Optogenetic mapping revealed that SC projections to the lateral posterior nucleus (LP) of the thalamus, a non-canonical polymodal sensory relay, are sufficient to mimic visually evoked fear responses. *In vivo* electrophysiology experiments identified a di-synaptic circuit from SC through LP to the lateral amygdale (Amg), and lesions of the Amg blocked the full range of visually evoked defensive responses. Our results reveal a novel collicular–thalamic–Amg circuit important for innate defensive responses to visual threats.

## Abstract (web summary)

The ability of animals to respond to life-threatening stimuli is critical for survival, yet the neural circuits mediating innate defensive behaviors are not well understood. Here, the authors reveal a novel collicular–thalamic–amygdala circuit critical for innate defensive responses to visual threats.

## Graphical abstract

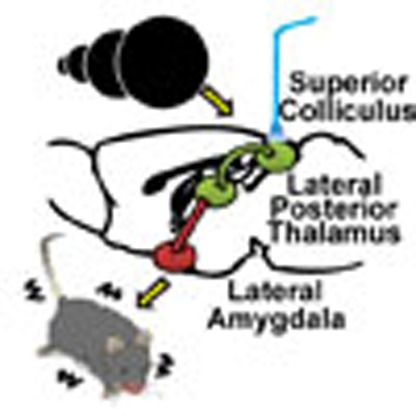

Defensive reactions to threat stimuli are a basic survival mechanism in all vertebrates^1^. It is known that the ability of prey animals to recognize predators is highly conserved and innate, even in naïve rodents^2^ and primates^3^. The visual system in particular is crucial for detecting potential threats, such as a shadow approaching from above, which have been simulated in behavioural experiments by presenting an expanding dark disc known as a looming stimulus^4^,^5^. Innately threatening visual stimuli activate modular circuits consisting of phylogenetically ancient brain structures^6^; however, details regarding the cell-type-specific circuitry mechanisms responsible for processing innate threat information remain to be elucidated. In particular, it remains unknown how visual information gains access to the amygdale (Amg), a structure shown to be essential for defensive responses to a wide range of threats^7^.

Several lines of behavioural evidence indicate that processing innately threatening stimuli in mammals may not require the participation of the cortical visual system. Visually guided danger recognition has been investigated in lower animals with limited visual cortical function^8^,^9^, in human neonates whose cortical networks are not fully developed^10^,^11^ and in experimental animals with visual cortical lesions^12^,^13^. Classical animal fear circuits demonstrated that parallel cortical and subcortical input routes exist, both of which can carry the auditory^14^,^15^ or visual^16^ conditioned stimuli to support fear conditioning. Further, human studies also revealed that patients with cortical blindness can still detect unconscious fearful signals^6^,^17^,^18^ through a presumed subcortical pathway that leads from the superior colliculus (SC) to the pulvinar of the visual thalamus (Pulv) and then to the Amg. However, no experimental evidence exists to support this hypothesis and controversy exists about the precise relay pathway that might connect the SC with the Amg^19^,^20^.

The SC is a subcortical center that mediates early sensorimotor integration and transformation, and many cells in the intermediate and deep layers of the SC (DLSC) were found to respond to various modalities of threatening stimuli^21^,^22^,^23^. Direct electrical or chemical stimulation of the intermediate or DLSC innately initiates a broad spectrum of defensive behaviours, both in rats^24^,^25^ and in monkeys^26^, and SC lesions also impair visually guided defensive behaviours^27^,^28^. Anatomical studies showed that in primates, the SC sends projections to the Pulv^29^,^30^, while in rodents the SC sends projections to the lateral posterior nucleus of the thalamus (LP, Pulv-like structure)^31^,^32^,^33^ and these areas subsequently project to high-order visual cortical areas^34^, the striatum^29^ or the lateral Amg (LA)^35^,^36^. Emerging evidence suggests that the Pulv, particularly its medial nucleus, is functionally important for fear emotion processing^37^, and a more recent study revealed that cells in the monkey medial Pulv rapidly and selectively respond to innate stimuli of snake images^38^. These findings raise the possibility that innate fear-related defensive reactions may depend on this subcortical pathway^19^.

In the present study, we aimed to examine the cell-type-specific circuit connectivity of the subcortical route for visual processing and determine the role of this pathway in mediating innate fear-related defensive behaviours. We first identify a sub-population of neurons in the medial region of the intermediate layers of the SC (ILSCm) that mediates the innate defensive response of mice to overhead looming stimuli (LS). Then, we optogenetically dissect a subcortical pathway from the glutamatergic projecting neurons in the ILSCm to the LP, which can be activated to initiate stereotyped long-lasting freezing behaviours (motion suppression and bradycardia). Retrograde trans-synaptic viral tracer labelling reveals that the LP serves as a key intermediate relay between the ILSCm and the LA. To the best of our knowledge, this is the first study to report very fast neural activation from the ILSCm to the LA (fastest <6 ms) through the LP. Furthermore, we show that the sustained network activation of the LA mediates the expression of the ILSCm-induced innate fear-related defensive behaviours.

## Results

### ILSCm is necessary for defensive responses to LS

**Figure 1:**
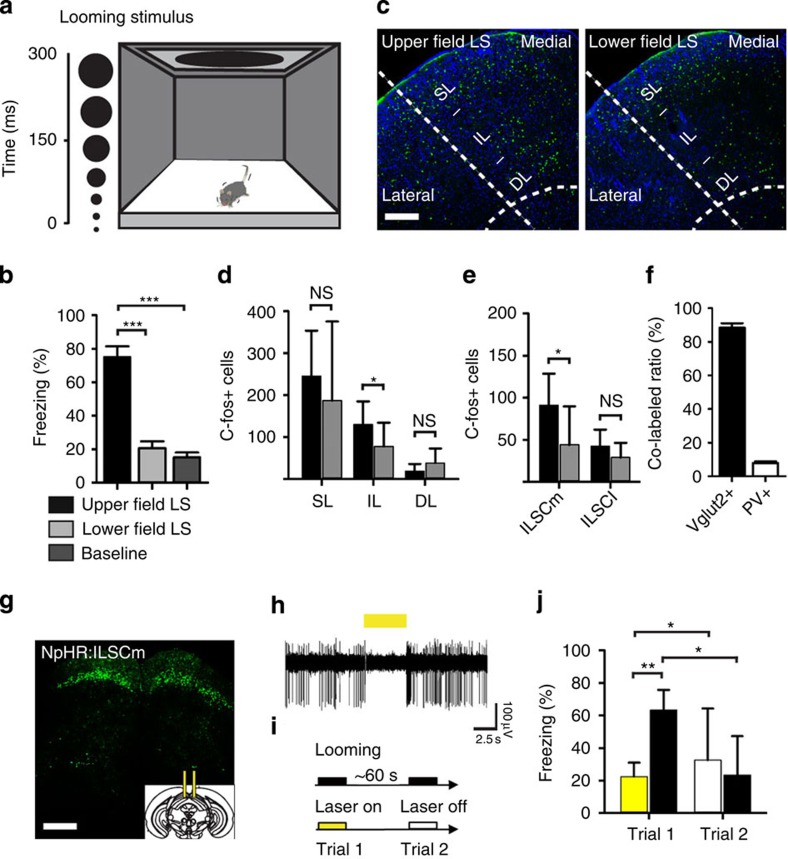
ILSCm glutamatergic neurons respond to upper field LS and mediate the LS-triggered innate defensive responses. (**a**) Schematic of the looming animation and testing environment. (**b**) Level of freezing during the 30-s period after stimulus onset. (*n*=7–8 subjects per group; ***P<0.001 by one-way ANOVA with Holm–Sidak *post-hoc* test) (**c**) Confocal images of the SC stained for c-fos 30 min after exposure to upper (left) or lower (right) field LS. The superficial layers (SL) included the superficial grey (SGS) and optic layer (SO); the intermediate layers (IL) included the intermediate grey (SGI) and intermediate white layer (SAI); and the deep layers (DL) included the deep grey (SGP) and deep white layer (SAP). The lateral or medial subdivision of the SC is approximately divided by the horizontal meridian in the collicular map. (green=c-fos; blue=DAPI). (**d**,**e**) Comparison of group difference of the c-fos levels in different layers of the SC (**d**) or in the medial and lateral ILSC (**e**) (*n*=24 slices per group; *P<0.05, NS *P*>0.05 by two-way ANOVA with Holm–Sidak *post-hoc* test) (**f**) Cell-type specificity of c-fos-expressing cells. Vglut2+, vesicular glutamate transporter 2 positive; PV+, parvalbumin positive. (**g**) eNpHR3.0–EYFP expression in CamKIIa neurons in the bilateral ILSCm. The inset shows the schematic of the implanted dual fibres. (**h**) *In vivo* electrophysiological identification of optogenetic inhibition of ILSCm neuron activities. (**i**) Experimental timeline of the optogenetic inhibition of the ILSCm during upper field LS. (**j**) Levels of freezing elicited by LS combined with (yellow) or without (white) optogenetic inhibition of the ILSCm. Black bars represent the EYFP control group. (*n*=7–8 subjects per group; **P<0.01, *P<0.05 by two-way ANOVA with Holm–Sidak *post-hoc* test). Values are represented as mean±s.d.; Scale bars: (**c**) 250 μm, (**g**) 500 μm.

SC responses to LS have been reported recently^39^; here we asked which specific group of neurons in the SC also participates in processing visually guided innate defensive behaviour. Overhead LS have been shown to trigger fleeing-to-nest or freezing responses in mice^9^. We focused on understanding the neural mechanisms of the LS-elicited freezing behaviours. A few early behavioural studies have distinguished this freezing behaviour from other SC-guided orientation and avoidance behaviours, and considered it as a more particular defensive response to a potential threat^24^,^40^. We found that when the protective nest was removed from the testing chamber (Fig. 1a), upper field LS elicited a robust unconditioned freezing (UF) response in all animals (*n*=8, 5 animals showed immediate freezing and the other 3 animals escaped to the corner and then froze) compared to the lower field LS control group (*n*=7) or baseline control group (without any LS; *n*=7; one-way analysis of variance (ANOVA), *F*(2, 20)=49.997, *P*<0.001). Holm–Sidak *post-hoc* tests revealed significantly more freezing in response to upper field LS than lower field LS (*P*<0.001) or baseline (*P*<0.001) (Fig. 1b). Moreover, repeated applications of the LS from above resulted in a rapid behavioural adaptation (Fig. 1j). Subsequent to behavioural testing, the level of activity-dependent c-fos expression in the SC was examined (Fig. 1c). As previously reported^39^, the highest number of activated cells in response to the LS was found in the superficial layers of the SC (SLSC), a moderate number in the ILSC and a small number in the DLSC (Fig. 1d). A two-way ANOVA on group (upper or lower field LS) and layers (SL, IL or DL) failed to reveal a statistically significant interaction [*F*(2, 143)=3.028, *P*=0.052]. Interestingly, Holm–Sidak *post-hoc* showed that the level of c-fos expression in the upper and lower field LS groups only differed in the IL condition (*P*<0.05), rather than SL (*P*=0.27) or DL (*P*=0.32) condition (Fig. 1d). Moreover, another two-way ANOVA for c-fos level in the IL on group and side (medial or lateral) found a significant interaction [*F*(1, 95)=5.094, *P*<0.026] and Holm–Sidak *post-hoc* showed that the only significant difference between groups was within the medial side (*P*<0.05), rather than the lateral side (*P*=0.057) (Fig. 1e), which is in accordance with the notion that this region spatially responds to aerial object stimuli^25^,^41^. Moreover, the ILSCm c-fos-positive population contained mainly glutamatergic neurons (Fig. 1f).

To examine whether these subpopulations of SC neurons were essential for mediating LS-induced defensive behaviours, an adeno-associated virus (AAV) carrying inhibitory eNpHR3.0–enhanced yellow fluorescent protein (eNpHR3.0–EYFP) under the control CaMKIIa promoter was bilaterally injected into the ILSCm (Fig. 1g). We found that CaMKIIa-positive neuronal somata were mostly localized at the upper surface of the ILSC, along the boundary of the stratum opticum and intermediate grey layer (Supplementary Fig. 1a,b). A continuous 588-nm laser was triggered during the LS to optogenetically inhibit the ILSCm CaMKIIa neurons (Fig. 1h,i). A two-way ANOVA for the level of freezing by trial (trial1 (laser on) or trial2 (laser off)) and group (NpHR:ILSCm (*n*=8) or EYFP:ILSCm control (*n*=7)) revealed a significant interaction (*F*(1, 29)=9.671, *P*<0.01). Holm–Sidak *post-hoc* showed that difference between groups in the trial1 (laser on) (*P*<0.01), and trial1 (laser on) was significantly lower than trial2 (laser off) within the NpHR group (*P*<0.05), indicating that silencing of ILSCm CaMKIIa neurons reversibly blocked the expression of LS-elicited freezing (Fig. 1j and Supplementary Movie 1). These results indicate that ILSCm CaMKIIa neurons play an essential role in mediating visually evoked innate defensive behaviours.

### Optical activation of ILSCm is sufficient to elicit freezing

**Figure 2:**
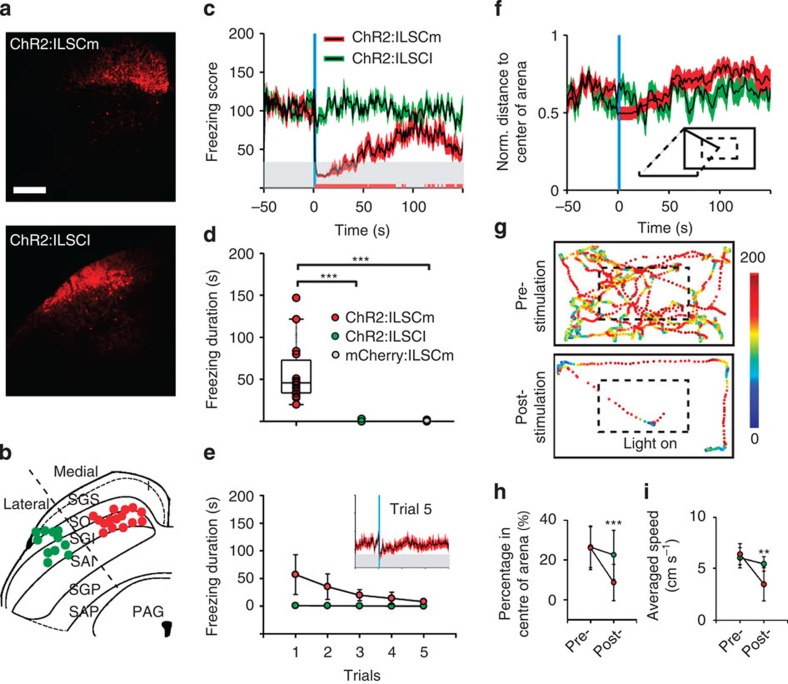
Optogenetic activation of ILSCm elicits unconditioned freezing behaviour. (**a**) ChR2-mCherry expression in CamKIIa neurons in the ILSCm or ILSCl. (red=mCherry; for number of ChR2+ cells in different layers, see Supplementary Fig. 1c). (**b**) Cannula tips for ChR2:ILSCm and ILSCl mice are indicated by the red and green dots, respectively. (**c**) Time courses of the averaged FS revealed that the ChR2:ILSCm (red) US (blue rectangle) elicited UF behaviour when compared against ChR2:ILSCl (green). Grey shaded area, animals are still in freezing states; the bottom red bars, the periods with significant group FS differences (*n*=15, 11 subjects for each group; *P*<0.05 by *t*-test). (**d**) Box plots of UF durations elicited by the US. (*n*=15, 11, 8 subjects for each group; ***P<0.001 by Kruskal–Wallis one-way ANOVA with Dunn's *post-hoc* test) (**e**) UF elicited by the US adapts significantly after repeated trials (red dots) (*n*=15 subjects, main effect *P*<0.001 by repeated one-way ANOVA). Inset, the time courses of the averaged FS before and after the fifth US. (**f**) Time courses of the normalized distance to the centre of the arena reveal that ChR2:ILSCm mice prefer the periphery of the arena after the UF has stopped. (**g**) Spatial FS map in the pre- or post-US period (3 min) from a sample ChR2:ILSCm mouse. The ‘light on' symbol indicates the onset of the US. Time spent in the centre of the arena (**h**) and the moving rate of the tested animals (**i**) during the post-UF period (subtracted the UF period from the post-US period). (*n*=15, 11 subjects for each group; ***P<0.001, **P<0.01 by two-way ANOVA with Holm–Sidak *post-hoc* test). Values are represented as mean±s.d.; Scale bars: (**a**) 250 μm.

Previous reports have shown that stimulation of different locations or populations of SC neurons results in a complex set of behaviours^23^,^24^,^25^,^26^, from defense-like responses to orienting responses. Here we optogenetically investigated the specific contribution of ILSCm CaMKIIa neurons. AAV virus carrying excitatory CaMKIIa–ChR2–mCherry was selectively injected into the ILSCm or into the lateral region of the ILSC (ILSCl) as a control (Fig. 2a,b). To mimic an unconditioned stimulus (US) on a freely exploring mouse, a phasic train of 473-nm light pulses (50 pulses at 20 Hz with a 2-ms pulse width) was manually triggered and delivered into the virus-targeting SC region. Note that the stimulation frequency was chosen to mimic the firing rates of natural visual responses in ILSC neurons (Supplementary Fig. 2c,f). Dramatically, light stimulation of the ChR2-expressing neurons in the ILSCm (ChR2:ILSCm, *n*=15), but not of the ChR2:ILSCl (*n*=11), immediately suppressed exploration on US onset and initiated a stereotyped ‘freezing' status that lasted from 20 to 150 s (Fig. 2c and Supplementary Movie 2). To further quantify this behavioural response, the movement trajectory of each animal was tracked to determine a ‘freezing score' (FS) parameter (lower score represents a higher level of freezing, Fig. 2c). The ChR2:ILSCm mice exhibited UFs with an average latency of 895±304 ms and the post-US FS was generally lower than the post-US FS in ChR2:ILSCl mice (Fig. 2c). A Kruskal–Wallis one-way ANOVA comparing the durations of UF across groups showed a significant difference (*χ*^2^(2)=25.325, *P*<0.001) and Dunn's *post-hoc* analyses revealed that the ChR2:ILSCm group (57±36 s) froze significantly longer than ChR2:ILSCl (*P*<0.001) or CaMKIIa–mCherry:ILSCm control groups (*n*=8) (*P*<0.001) (Fig. 2d). Another important feature of freezing responses to threat stimuli is bradycardia activity^42^,^43^. Thus, we also continuously monitored the heart rate (HR) of ChR2:ILSCm mice (*n*=8) and found that the HR was immediately reduced by an average of 10% after the US onset and remained below the baseline level for the duration of the freezing period (Supplementary Fig. 3a,b).

Stimulation of ILSCm CaMKIIa neurons induced subsequent behavioural responses; after the animal stopped freezing it quickly cowered in the corner of the arena to avoid entering the open spaces and showed sustained immobility. Two-way ANOVAs on time point (pre or post UF for 3 min) and group (ChR2:ILSCm or ChR2:ILSCl) found significant interactions for both time spent in the centre of the arena (*F*(1, 51)=4.63, *P*<0.05) and speed (*F*(1, 51)=12.715, *P*<0.001) (Fig. 2f–i). These behaviours have previously been reported to reflect the innate anxiety and fear responses of animals to predator cues^44^,^45^. To further test whether the stimulation affects an animal's normal motor function, we delivered an US to the ChR2:ILSCm mice when a protective nest was available (*n*=6) (Supplementary Fig. 3c). Under this condition, the mice still exhibited UF (Supplementary Fig. 3d) and then quickly fled into the nest and remained hidden in the nest for an average duration of 866±537 s (Supplementary Fig. 3e–g). In addition, repeated applications of the US to the ILSCm at 3-min intervals also resulted in the reduction of the total UF time (repeated one-way ANOVA, *F*(4, 66)=15.293, *P*<0.001) (Fig. 2e and Supplementary Movie 2), similar to the effects elicited by repeated LS (Fig. 1h), reflecting rapid adaptation of this innate defensive behaviour.

We found that the freezing elicited by the US in the ChR2:ILSCm was not directly dependent on the activation of the adjacent DLSC and periaqueductal grey (PAG), which are generally considered as the major targeting regions of defense responses^23^,^46^. First, these regions showed only sparse mCherry-expressing axon fibres under the current virus dosage (Supplementary Fig. 1a,b). Moreover, a supplementary test showed that activation of the DLSC–PAG pathway (*n*=3) elicited wild running or backward fleeing behaviours, similar to the behaviours found by directly stimulating the PAG^45^, rather than the immediate freezing behaviour (Supplementary Movie 3).

We also examined the potential involvement of gamma-aminobutyric acid (GABA) ergic cells in the SC. Due to the non-specificity of the expression, about 5% of ILSCm CaMKIIa cells were found to be parvalbumin (PV) positive (Supplementary Fig. 1c–e). Application of the optical US to the ILSCm in transgenic Vgat–ChR2–EYFP mice (*n*=7) did not elicit freezing. On the other hand, when ChR2 was expressed in ILSCm vesicular glutamate transporter 2 (Vglut2) neurons using a combination of Vglut2–ires–cre mice and the Cre-dependent ChR2 virus method, the application of the US to the ILSCm Vglut2 neurons also elicited long-lasting freezing responses with average durations of 38±9 s (*n*=7) (Supplementary Fig. 4).

In summary, we have shown that 20-Hz phasic light stimulation of ILSCm CaMKIIa neurons (or a more specific population of Vgult2 neurons) evokes a stereotypical prolonged innate freezing behaviour. This response, along with the subsequent anxiety and avoidance responses, indicates that the stimulation led to sustained activation of the brain's aversive system^1^.

### Amg is necessary for ILSCm-elicited defensive responses

**Figure 3:**
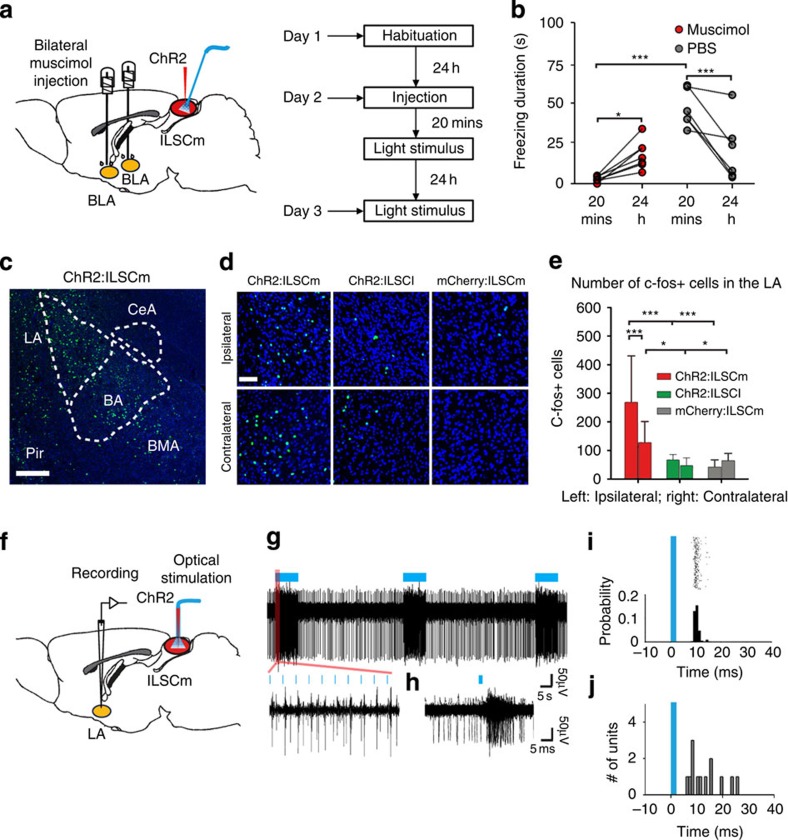
Activation of the amygdala is necessary for the UF elicited by the US in the ILSCm. (**a**) Left, schematic of the drug injections into the bilateral BLA via cannulas and fibre optic implantations in the ILSCm. Right, examination timeline of the impact of BLA on the UF, elicited by application of the US on the ILSCm. (**b**) The duration of UF elicited by application of the US in the ILSCm after BLA infusion (muscimol or PBS) at 20 min and 24 h (repeated test) (*n*=6–7 subjects per group; ***P<0.001, *P<0.05 by two-way ANOVA with Holm–Sidak *post-hoc* test). (**c**) A low-magnification image of the amygdala, stained for c-fos, 30 min after applying the US to the ILSCm. The c-fos-positive cells are mainly located in the LA (green=c-fos; blue=DAPI). Pir, piriform cortex; BA, basal nuclei of BLA; CeA, central amygdala; BMA, basomedial amygdala. (**d**) High-magnification images of c-fos-positive cells in the bilateral LA. (**e**) The c-fos levels in the bilateral LA (*n*=18 slices per group; ***P<0.001, *P<0.05 by two-way ANOVA with Holm–Sidak *post-hoc* test). (**f**) Schematic of the stimulation experiments in the ILSCm and simultaneous recording in the LA. (**g**) Activation of an LA neuron during optogenetic stimulation of the ILSCm (top) and a magnified plot showing 10 light pulses (bottom left). (**h**) This LA neuron is orthodromically activated by ILSCm pulsed-laser stimuli (blue). (**i**) Peristimulus time histogram (PSTH) of the example LA neuron reveals the distribution of response times to the upstream ILSCm activation (blue=light, peak response latency≈9 ms). (**j**) Histogram of the peak response latencies to the onset of light pulses (blue) for 13 identified LA neurons. Values are represented as mean±s.d.; Scale bars: (**c**) 250 μm; (**d**) 50 μm.

Next, we studied the neural circuitry from the ILSCm that mediates the innate defensive behaviours. The Amg is a critical structure for both conditioned^47^ and unconditioned^48^ fear responses; thus, we tested the function of the Amg as the potential centre mediating ChR2:ILSCm US-elicited responses. A GABA_A_-receptor agonist (muscimol) was bilaterally injected to reversibly inactivate the basolateral complex of the Amg (BLA) (*n*=7) (Fig. 3a and Supplementary Fig. 5); phosphate-buffered saline (PBS) was injected into the BLA in a control group (*n*=6). None of the ChR2:ILSCm mice showed US-elicited UF when the US was delivered after 20 min of BLA inactivation (Fig. 3b). About 24 h later, after the muscimol had washed out, the US was again effective at eliciting UF with an average duration of 18.0±8.8 s (Fig. 3b and Supplementary Movie 4). A two-way ANOVA for the duration of UF using time post infusion (20 min or 24 h) and treatment (muscimol or PBS) found a significant interaction (*F*(1, 25)=19.303, *P*<0.001). Holm–Sidak *post-hoc* tests showed that the duration of UF was significantly higher by 24 h as compared with 20 min after muscimol infusion (*P*<0.05), and there was only a significant difference in drug treatments at 20 min (*P*<0.001), but not 24 h following drug infusion (Fig. 3b). These results suggest that ILSCm US-elicited defensive behaviours require BLA activation.

To confirm that Amg activation indeed occurred during the UF behaviour, the level of c-fos in the Amg after the application of the US was examined (Fig. 3c,d). A two-way ANOVA for c-fos expression levels in the LA revealed a significant interaction between group (ChR2:ILSCm, ChR2:ILSCl or mCherry:ILSCm) and hemisphere (ipsilateral or contralateral to the stimulation site) (*F*(2, 83)=11.530, *P*<0.001). Holm–Sidak *post-hoc* showed that the c-fos levels in the bilateral LA for the ChR2:ILSCm group were significantly higher than the other two groups (ipsilateral LA: *P*<0.001, contralateral LA: *P*<0.05), and the ipsilateral LA has higher c-fos expression than the contralateral LA only for the ChR2:ILSCm group (*P*<0.001) (Fig. 3e).

We investigated whether a functionally efficient information transmission route from the ILSCm to the LA existed by utilizing a combination of optogenetics and *in vivo* electrophysiological recording methods. A 20-Hz pulsed-laser light was delivered to the ChR2-expressing ILSCm region and the electrical activity in the ipsilateral LA was recorded in anesthetized mice (Fig. 3f and Supplementary Fig. 6a,b). It was found that 13/38 LA cells responded to the ILSCm stimulation (Fig. 3g,h and Supplementary Fig. 6c) and that the latency of the peak response ranged from 6 to 26 ms with an average of 13.3±6.6 ms (Fig. 3i,j). This result shows for the first time that ILSCm CaMKIIa neurons can send a rapid signal to the downstream LA.

**Figure 4:**
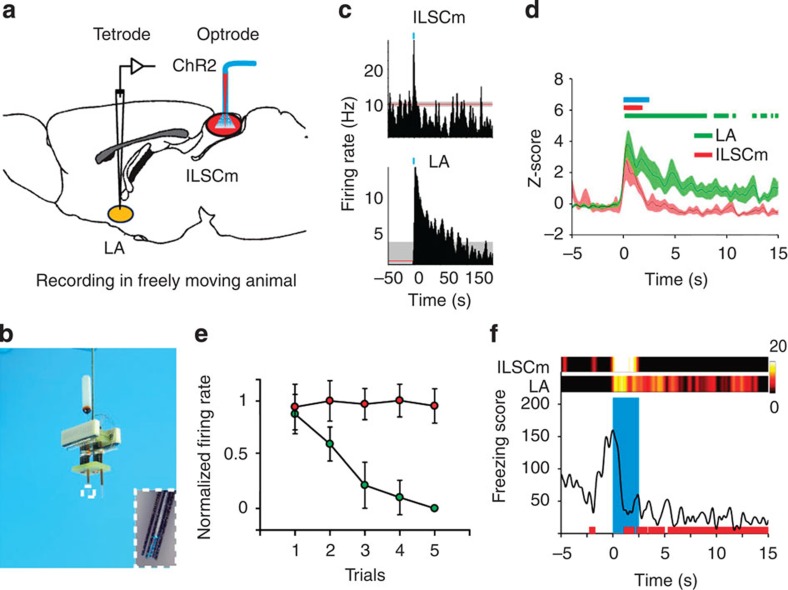
Activation of the LA is temporally correlated with the UF elicited by applying the US to the ILSCm. Schematic (**a**) and photograph (**b**) of the two-site multi-channel optrode system. The inset in **b** shows the tip of the optrode (white box). (**c**) PSTHs of two example neurons from the ILSCm and LA show different firing patterns in response to the US in the ILSCm (1-s bins). The red line represents the estimation of the expected baseline rate and the grey shaded area represents the confidence limit of 99%. (**d**) *Z*-scored population PSTH of responsive neurons from the ILSCm (red) and LA (green) (100-ms bins), shaded areas represent the s.e.m. The blue bar indicates the optical stimulation, red bar indicates the period of excitation of ILSCm neurons (*n*=7 cells, compared with the pre-event reference period, *P*<0.01 by right-tailed *t*-test), and green bar indicates the period of excitation of LA neurons (*n*=11 cells). (**e**) The normalized post-stimulation mean firing rates of responsive LA neurons (green) show a significant adaptation after each trial (*n*=11 cells, main effect *P*<0.001 by repeated one-way ANOVA). Contrarily, the firing rates of responsive ILSCm neurons (red) did not adapt across trials (*n*=7 cells, main effect *P*=0.96 by repeated one-way ANOVA). (**f**) The ILSCm and LA example neuron activity (top; scaled by the hot colour map) were aligned with the FS with time (the bottom red bars represent the freezing state). Values are represented as mean±s.d.

In freely behaving mice, we aimed to identify the specific functional mechanism of the ILSCm-LA circuitry by which optical stimulation modulates the neural network activities. We developed a novel two-site multi-channel optrode system for simultaneous recording and light delivery in freely moving mice (Supplementary Fig. 7a) and applied this to stimulate and record in ILSCm, and simultaneous recording from LA (Fig. 4a,b and Supplementary Fig. 7e,f). In mice exhibiting prolonged UF elicited by the US applied to the ChR2:ILSCm mice (*n*=3), 20 and 40 single-unit spikes were recorded in the ILSCm and LA, respectively (Supplementary Fig. 7b,c). Under direct light-evoked responses, 7 of the 20 local ILSCm cells were found to fire immediately following the pulsed laser, with an average latency of 3.0±1.5 ms, and the cells showed transient excitation during the US (Fig. 4c,d). In addition, 11 of the 40 LA cells were activated by the pulsed-laser stimulation, with a latency distribution comparable to that found in anesthetized mice (Supplementary Fig. 7d,g), and these cells showed sustained post stimulation excitation (Fig. 4c,d). Intriguingly, LA neuronal activation showed adaptation in firing rates among the US trials, whereas ILSCm neuronal activation was invariant (Fig. 4e). By comparing the time courses of the physiological activities and behavioural responses, it was discovered that the activation of LA neurons, rather than that of ILSCm neurons, temporally correlated with the US-elicited UF in ChR2:ILSCm mice (Fig. 4f and Supplementary Fig. 8). These results suggest that activation of the ILSCm represents an ‘on switch' for the brain's defensive system, whereas sustained LA activation is responsible for prolonged freezing in animals.

In summary, we provided electrophysiological evidence that the inputs from the ILSCm reach the LA through a rapid shortcut route. Furthermore, at the behavioural level we showed that the LA mediated ChR2:ILSCm US-elicited defensive behaviours. Collectively, these data support the hypothesis that a subcortical pathway from the SC to the Amg is involved in the rapid visual processing of emotional stimuli^49^. However, this raises questions about the neuronal substrate of this presumed pathway, or more specifically, whether and how (if any) the Pulv-related thalamic structures in mice can relay signals from the ILSCm to the LA.

### LP projection is crucial for ILSCm evoked defensive behaviour

**Figure 5:**
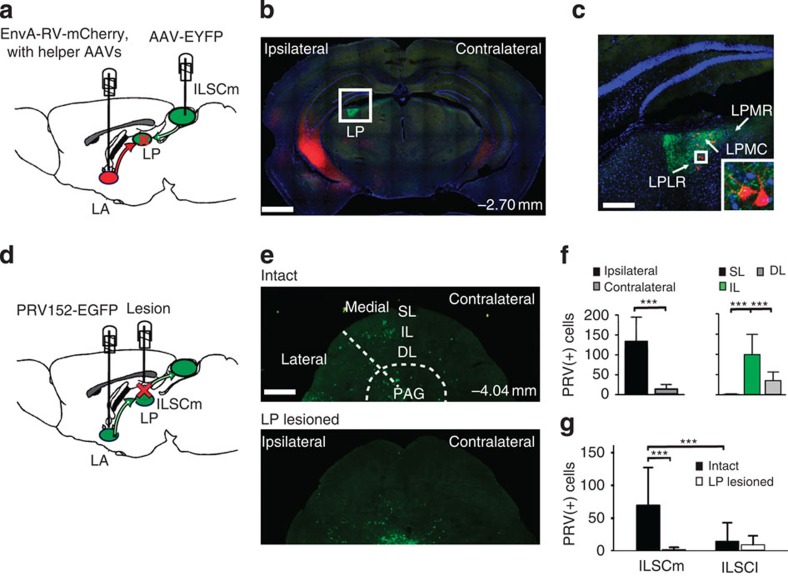
The lateral posterior nucleus of the thalamus is the critical monosynaptic relay underlying the ILSCm–LA circuit. (**a**) Schematic of AAV–EYFP-mediated anterograde tracing in the SC and EnvA–RV–mCherry in combination with helper AAV-mediated trans-monosynaptic retrograde tracing in the LA. (**b**) Coronal brain section shows the anterograde projection pattern from the SC (green) and the distribution of trans-monosynaptically labelled neurons from the LA (red). (**c**) A magnified view of the LP shows the co-localization of axon terminals from the SC projection neurons and the soma of the LP neurons that project to the LA. Inset white box depicts the area. (**d**) Schematics of the PRV152–EGFP injection in the LA and of the ibotenic acid injection in the LP. (**e**) Coronal sections show PRV(+) cells (green) in the SC from an intact (top) and LP-lesioned mouse (bottom). (**f**) PRV(+) cells are mainly located in the ILSCm (*n*=12 slices; ***P<0.001 by Kruskal–Wallis one-way ANOVA with Dunn's *post-hoc* test). (**g**) Comparison of the PRV(+) cells in the medial and lateral ILSC between intact and LP-lesioned mice (*n*=12 slices per group; ***P<0.001 by two-way ANOVA with Holm–Sidak *post-hoc* test). Values are represented as mean±s.d.; Scale bars: (**b**) 1 mm; (**c**) 250 μm; (**e**) 500 μm.

Anatomical evidence for subcortical pathway connections has been shown in rodents^16^,^31^, nonhuman primates^29^ and humans^49^. In this study, we aimed to identify the connections of the ILSCm–LA subcortical pathway and to determine its function in the innate defensive behaviours of mice. To test the possible circuitry by which the ILSCm and LA interact, we injected the anterograde tracer, AAV–CaMKIIa–EYFP, in the ILSCm and the retrograde trans-monosynaptic tracer, EnvA–rabies virus (RV)–mCherry, in the LA (Fig. 5a and Supplementary Fig. 9a–c). The ILSCm CaMKIIa axons prominently project to the LP, a mouse Pulv-like structure (Fig. 5b). Retrograde RV-positive cells raised from the starter cells in the LA were simultaneously found in the LP, as shown by co-labelling them with green fluorescent protein (GFP) from AAV and with mCherry from rabies (Fig. 5b,c and Supplementary Movie 5), which were mostly confined in the LR and MC subdivisions of the LP (Supplementary Fig. 9d).

Therefore, it appears that the LP is an important intermediary relay in the ILSCm–LA circuit. To further test this idea, the time-dependent retrograde and trans-synaptic pseudo-rabies virus (PRV)–EYFP was unilaterally injected into the LA (Fig. 5d). Forty-eight hours after PRV:LA infusion, PRV positive (+) cells were initially found in the ipsilateral SC, specifically located in the ILSCm (Supplementary Fig. 10a). By 60 h, PRV(+) cells were prominently expressed in the SC (Fig. 5e,f and Supplementary Fig. 10a,b), and a Kruskal–Wallis one-way ANOVA for PRV(+) cells found a significant different between the ipsilateral and contralateral SC (*χ*^2^(1)=17.925, *P*<0.001) (Fig. 5e). Moreover, PRV(+) cells were largely distributed in the ILSC, and a smaller number in the DLSC, with almost none in the SLSC (Kruskal–Wallis one-way ANOVA on different layers (SL, IL or DL), *χ*^2^(2)=29.573, *P*<0.001) (Fig. 5e). In addition, more PRV(+) cells were found in the ILSCm than in the ILSCl (Fig. 5g). Furthermore, inducing lesions on the LP cell bodies using ibotenic acid resulted in a marked reduction of PRV(+) cells. A two-way ANOVA for PRV(+) cells in the ILSC for treatment (LP lesioned or intact) and location (ILSCm or ILSCl) revealed a significant interaction (*F*(1,47)=16.972, *P*<0.001). Holm–Sidak *post-hoc* tests showed that the expression of PRV(+) cells was significantly reduced for the LP-lesioned group in the ILSCm (*P*<0.001) rather than ILSCl (*P*=0.426) (Fig. 5g and Supplementary Fig. 10d).

**Figure 6:**
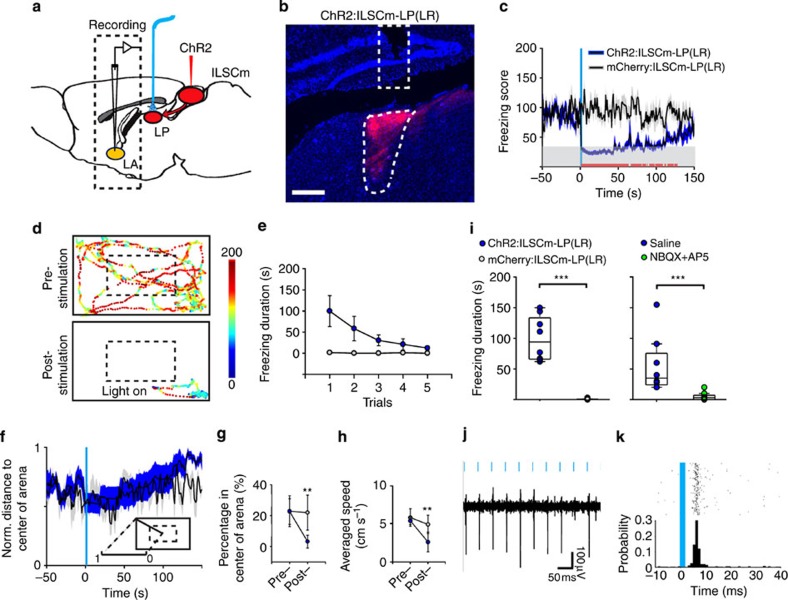
Activation of terminals in the LP that projected from the ILSCm-elicited fast responses in LA neurons and unconditioned freezing behaviour. (**a**) Schematic of analysing the function of ILSCm–LP pathway. A optic fibre was implanted in the ipsilateral LP of ChR2:ILSCm mice. In addition, a recording electrode was placed in the LA. (**b**) Confocal image of ChR2-positive terminals in the LP(LR) that from the ILSCm and the tip of optic fibre (the top dotted rectangle). red=mCherry; blue=DAPI. (**c**) Time courses of the averaged FS reveals that the US (blue rectangle) of ChR2:ILSCm–LP(LR) terminals elicits UF and a prolonged lower FS (blue) compared with the control (black) (*n*=8 subjects for per group; *P*<0.05 by *t*-test). (**d**) Spatial FS map of the pre- or post-US period (3 min) from a sample ChR2:ILSCm–LP(LR) mouse. The ‘light on' symbol indicates the onset of the US. (**e**) The UF elicited by the US of ILSCm–LP(LR) terminals adapts significantly after repeated trials (blue dots, *n*=8 subjects, main effect *P*<0.001 by repeated one-way ANOVA). (**f**) Time courses of the normalized distance to the centre of the arena reveal that animals prefer the periphery of the arena after they are relieved of the UF elicited by the US of ChR2:ILSCm–LP(LR) terminals. (**g**,**h**) Time spent in the centre of the arena (**g**) and the animal speed (**h**) show a significant reduction in the post-UF period for the US of ChR2:ILSCm–LP(LR) terminals (*n*=8 subjects for per group; **P<0.01 by two-way ANOVA with Holm–Sidak *post-hoc* test). (**i**) Box plots of the duration of UF elicited by the US. The glutamate receptor antagonists NBQX plus AP5 (D(-)-2-amino-5-phosphonovaleric acid) infusion in LP(LR) blocks the UF response (*n*=7–8 subjects per group; ***P<0.001 by Kruskal–Wallis one-way ANOVA with Dunn's *post-hoc* test). (**j**,**k**) A sample LA neuron is activated by the pulsed laser in the upstream ILSCm–LP(LR) terminals (peak response latency ≈5 ms, (**k**). Values are represented as mean±s.d.; (**b**) 250 μm.

Finally, we asked whether the US-elicited innate defensive behaviours in the ChR2:ILSCm depend on the activation of the ILSCm–LP pathway. An optic fibre was implanted in the LR subdivisions of the LP of CaMKIIa–ChR2:ILSCm mice (*n*=8) or mCherry:ILSCm control mice (*n*=8) (Fig. 6a,b). As expected, stimulating ILSCm–LP(LR) terminals with the US also elicited the typical UF behaviour with a mean duration of 100±37 s (Kruskal–Wallis one-way ANOVA, *χ*^2^(2)=9.975, *P*<0.001) (Fig. 6c,i), followed by post-UF open space avoidance and cowering (two-way ANOVAs for time spent in centre of arena (*F*(1, 29)=8.733, *P*<0.01) and speed (*F*(1, 29)=5.162, *P*<0.05)) (Fig. 6d,f–h), as well as rapid behavioural adaptation (repeated one-way ANOVA, *F*(4, 35)=26.412, *P*<0.001) (Fig. 6e). To further confirm that the behavioural effects of terminal stimulation were not due to passing depolarizing axons or to the back propagation of action potentials to ILSCm cell bodies (antidromic stimulation)^50^,^51^, the glutamate receptor antagonists NBQX plus AP5 (*n*=7) or saline control (*n*=8) were locally injected in the postsynaptic LP(LR) region before terminal stimulation. The response evoked by the US in ILSCm–LP(LR) terminals was blocked by glutamate receptor antagonists (Kruskal–Wallis one-way ANOVA, (*χ*^2^(2)=9.456, *P*<0.001) (Fig. 6i and Supplementary Movie 6). Moreover, c-fos expression in the LA (Supplementary Fig. 11a,b) and very short latency neural responses in the LA (9.9±6.0 ms, 13/61 cells) were also found in response to the US in ILSCm–LP(LR) terminals (Fig. 6j,k and Supplementary Fig. 11e–h).

In summary, our data demonstrate that LP forms monosynaptic connections with the ILSCm and LA and is a crucial relay for neural transmission from the ILSCm to the LA. Moreover, we revealed that the LR or MC subdivision of the LP is the downstream target when processing US-initiated freezing behaviour in the ILSCm.

## Discussion

In the present study, we found that the subcortical ILSCm–LP–LA pathway mediates visually evoked innate fear-related defensive freezing behaviour in mice. A population of glutamatergic neurons in the ILSCm responded to and mediated an unlearned freezing response to a looming stimulus in the upper visual field. For rodents, many natural predators approach from above and are detected in the upper visual field. Behavioural evidence indicates that rodents have a continuously overlapping upper visual field representation that facilitates their alertness to aerial predators^52^. The upper visual field signal is represented in the medial region of the SC's spatial map^25^. Thus, ILSCm neurons may detect overhead motion through direct retinal projections^53^,^54^ and innately recognize this type of information as a potential threat without prior experience. To further test this idea, a phasic laser pulse train was delivered to ChR2-expressing glutamatergic neurons to mimic an unpredicted visual input to the ILSCm. This type of optogenetic stimulation initiated long-lasting freezing behaviour followed by a sustained increase of innate fear and anxiety responses. Next, *in vivo* recording revealed that the ILSCm rapidly transmits (fastest <6 ms) signals to the LA (Fig. 3i,j) and that activation of the LA is crucial for defensive behaviours elicited by ILSCm stimulation. The ILSCm sends direct projections to the LP, and a very recent study also indicated that a subset of SC–LP projecting neurons was sensitized to small, moving objects^33^. Trans-synaptic tracer labelling showed that the LP is the key relay nuclei that connects the ILSCm and the LA. Activating the subcortical ILSCm–LP glutamatergic input pathway evoked similar short latency responses in the LA and elicited identical innate freezing responses.

The SC is largely known to mediate a complex set of defensive behaviours though its extensive descending projections to limbic structures, the basal ganglia and the brainstem. In this study, we dissected the contribution of a specialized ILSCm–LA pathway in mediating a stereotyped, innate defensive freezing behaviour. The US input signal initiated in the ILSCm rapidly reaches the LA via a crucial relay in the LP nucleus. Other possible subcortical or cortical routes originating from the SC may not be directly involved in this type of innate behaviour. First, the loops between the ILSC/DLSC and the basal ganglia, via the substantia nigra, have been shown to detect salient events^55^ and mediate motivational behaviours such as orientation or saccades, which are important components of defensive behaviours^56^. However, the descending pathway from the SC to the substantia nigra originates in the lateral, rather than in the medial part of the deep layers^55^. Second, the ipsilateral efferent bundles from the ILSC/DLSC also send projections to regions of the brainstem, such as the PAG and the cuneiform nucleus. The PAG is commonly known to be a major output nucleus of the defense system. Moreover, recent studies have found that the PAG may also transmit signals about unconditioned painful stimuli to the LA to support fear conditioning or innate responses^45^,^57^. Thus, in this study, we avoided directly activating the DLSC–PAG terminal during the ILSCm somata US experiments. Also, a control test showed that the innate responses elicited by DLSC–PAG pathway activation are more similar to those resulting from PAG stimulation^45^ than those resulting from ILSCm stimulation. The SC–cuneiform nucleus pathway most likely plays a role in defensive or fear-related cardiovascular responses^58^,^59^, which are beyond the scope of the present discussion. Finally, the LP can transmit signals from conditioned visual stimuli to the LA, not only via direct subcortical projections, but also via indirect (that is, LP–temporal association area/perirhinal cortex–LA) routes^16^. Results from a supplemental experiment (Supplementary Fig. 11c,d) examining c-fos levels in the temporal association area/perirhinal cortex areas after ILSCm–LP stimulation showed that these two regions were minimally activated.

The current results demonstrate that the ILSCm–LP–LA pathway constitutes an innate circuit for detecting and reacting to simple looming threat stimuli, supporting the hypothesis that the subcortical pathway primarily carries low resolution, but important visual information for survival^60^. Considering previous debates^19^,^20^ concerning the existence of this simple emotion processing route, our current findings shed new light on several areas. First, it is more likely that the intermediate layers, rather than the superficial layer of the SC^16^,^31^, connect to the LA through some subdivisions of the LP. This idea challenges the standard hypothesis of the subcortical pathway, for which it is generally proposed that low-frequency visual input signals arrive at the SLSC and are transmitted to the inferior Pulv. Alternatively, the ILSC neurons can receive the excitatory input from the SLSC^61^ or even direct afferents from the direction-selective retinal ganglion cells^54^. Although the specific role of the ILSCm neurons in encoding the innate visual stimuli still needs to be further investigated, overhead looming-stimulus-responsive ILSCm neurons were recorded in this study. More importantly, the ILSC sends projections to the medial, rather than the inferior, Pulv in monkeys^30^, and only the medial Pulv subsequently projects to the Amy^62^. Thus, the ILSC–medial Pulv–Amy pathway could be a more reasonable model than the standard SLSC–inferior/medial Pulv–Amy pathway model, due to the lack of intrinsic connections between different Pulv subnuclei. In addition, the trans-synaptic tracer labelling result in this study also indicated the SLSC were not retrogradely targeted by the LA cells. In rodents, the LP is a Pulv-related structure^63^ and is segregated from the Pulv in monkeys^64^. Similar to the Pulv, the LP has been divided into several subdivisions^63^ and also receive different SC inputs^35^,^65^. Our results showed that the LP(LR) and LP(MC) could be the monosynaptic relays between the ILSCm and the LA, nevertheless, whether this areas corresponded to the medial Pulv still needs to be further clarified. The rodent LP was suggested to play a central role in spatial attention and neglect, which are also closely related to Pulv functions. In this study, we showed that the ILSCm–LP pathway specifically mediates innate defensive freezing behaviour in mice. This finding has striking parallels with a recent study in monkeys showing that Pulv neurons can selectively detect visual images of an unreal predator threat (that is, pictures of snakes)^38^.

The current study on the subcortical innate fear circuit in rodents may lead to new implications regarding the circuit mechanisms that underlie mental disorders in humans. First, the activation of the innate fear-related defensive behaviours via the ILSCm–LP–LA pathway generated a more sustained anxiety state in mice (Fig. 2h,i), supporting the notion that human fear and anxiety might involve the reactivation of innate fear circuits^46^,^66^ and that constant over-activation of this pathway may underlie anxiety disorders or post-traumatic stress syndrome. Moreover, it has been suggested that impairments in emotional and social cognition, which are recognized as major symptoms in patients with schizophrenia and autism, are caused by the reduction of Amg activation during emotional (fearful) stimuli^67^,^68^. Notably, individuals with schizophrenia and autism exhibit eye movement disturbances^69^,^70^, which may involve the function of the SC. Therefore, a reduction in the ability of the Amg to discriminate threat from natural stimuli may originate from abnormal signalling in the subcortical pathway.

## Methods

### Animals and viral expressions

Adult (6–8 weeks) male C57BL/6, Vgat–(ChR2(H134R))–EYFP and Vglut2–ires–cre mice were used in the experiments. For optogenetics, AAV5 viruses encoding CaMKIIα–hChR2(E123T/T159C)–mCherry, CaMKIIα–eNpHR3.0–EYFP or DIO–hChR2 (H134R)–mCherry were packaged. For virus-mediated retrograde tracing, trans-multiple synaptic tracer CMV–PRV-152–EGFP and trans-monosynaptic tracer EnvA–RV–mCherry were prepared. CaMKIIα-eNpHR3.0 virus (0.7 μl) was injected into the bilateral ILSCm (AP: −3.70 mm; ML:±0.60 mm; DV: −1.85 mm), CaMKIIα–hChR2 virus was injected into the unilateral ILSCm or the ILSCl (AP: −3.70 mm; ML: 1.30 mm; DV: −2.20 mm) of the C57BL/6 mice and DIO–hChR2 virus was injected into the unilateral ILSCm of Vglut2–ires–cre mice. Electrophysiological or behavioural experiments were performed at least 4 weeks post injection. For terminal stimulation, the duration of viral incubation lasted an additional 2–4 weeks. Animal husbandry and all aspects of experimental manipulation of the animals were approved by Animal Care and Use Committees at the Shenzhen Institute of Advanced Technology (SIAT) or Wuhan Institute of Physics and Mathematics (WIPM), Chinese Academy of Sciences (CAS).

### Implantation of optical fibre(s) and drug cannulae

For optical stimulation of the SC somata, an implantable optic fibre (NA=0.37, *Φ*=200 μm) was inserted unilaterally into the ILSCm (AP: −3.70 mm; ML: 0.50 mm; DV: −1.40 mm) or the ILSCl (AP: −3.70 mm; ML: 1.25 mm; DV: −1.80 mm). The fibre was implanted for SC–LP terminals stimulation (AP: −2.40 mm; ML: 1.50 mm; DV: −2.10 mm). For yellow light stimulation of dual ILSCm, the fibres were implanted at a 20° angle from the vertical axis (AP: −3.70 mm; ML: ±1.25 mm; DV: −1.50 mm). For optogenetic stimulation with drug application, the fibre was implanted at 15° from the vertical axis (AP: −4.10 mm; ML: 0.50 mm; DV: −1.45 mm). Guide cannulae (OD=0.48 mm, with dummy screw caps) were implanted bilaterally to inject into the BLA (AP: −1.70 mm; ML:±3.20 mm; DV: −3.60–3.70 mm), or unilaterally in the LP (AP: −2.40 mm; ML: 1.50 mm; DV: −1.60 mm).

### Behavioural experiments and optical stimulation

The looming stimulation test was performed in a 40 × 40 × 30-cm closed box. An LCD monitor was embedded into the ceiling to present the looming stimulus. The looming stimulus, which consisted of an expanding black disc, appeared at a diameter of 2° to 20°, and was repeated 15 times in 5 s. No shelter nest was placed in the box and the stimulation was triggered by the experimenter manually. For the optogenetic inhibition experiment, two implanted optic fibres were connected to a 588-nm yellow light laser and the light power was set to about 15–20 mW at the fibre tips. The light was delivered into the SC simultaneously with the onset of the looming stimulus, and the duration of neuronal inhibition fully encompassed the duration of the looming stimulus.

Optogenetic stimulation experiments were performed in a 40 × 40 × 30-cm rearing cage box. All animals were pre-handled before the tests and an additional habituation stage was performed for HR monitoring to acclimate the mice to a neck collar sensor or a removable dummy cap in case of the drug-infusion mice. For the optogenetics–stimulation experiment, the implanted optic fibre was connected to a 473-nm blue light laser and the light power was set to about 7–10 mW at the fibre tip for cell body stimulation and 15–20 mW for terminals stimulation. Five repeated US trials (50 laser pulses of 2 ms at 20 Hz) were delivered in the targeting regions. The inter-trial interval was set to ∼3 min and was controlled manually; the light stimulus was triggered only during mouse movement.

For the Amg inactivation experiments, the GABA receptor agonist muscimol dissolved in 0.2 μl PBS was infused at 0.1 or 0 μg (control) per hemisphere into the bilateral BLA. For the LP glutamate receptors blockade experiment, a 0.3 μl mixture of 10 mM NBQX and 30 mM AP5 dissolved in 0.9% saline or a saline-only control was infused into the LP. Drug infusions were performed during the 20 min before the stimulation test and 24 h later, after the drug was fully metabolized, the mice were retested following the same method.

Animal behaviours were recorded by cameras and analysed offline using Anymaze. Freezing behaviour was evaluated by a FS parameter; a smaller FS value represents lesser movement. To describe the open space avoidance of mice, an additional parameter, that is, the distance to the center was calculated: the center of the arena was set as the zero point and the positional deviation of the animal to the zero point was measured and normalized.

### Anesthetized optical stimulation and electrophysiological recording

Mice were anesthetized with urethane and secured in a stereotaxic apparatus. An optic fibre was placed in the ILSCm (AP: −4.1 mm; ML: 0.5 mm; DV: −1.45 mm at a 15° angle from the vertical axis) and connected to a blue light laser. A bundle of 8 stereotrodes was inserted in the LA (AP: −1.7 mm; ML: 3.2 mm; DV: 3.5–4.5 mm below the dura) and single-unit activity was collected by the Plexon System (Plexon, Dallas, USA). Pulsed laser (20 Hz with 2 ms width) was delivered to the ILSCm when stable spontaneous spikes of the LA neurons were detected. At the end of the experiment, recording sites were marked through electrolytic lesions. Mice were sacrificed and the brain tissue was processed histologically to verify the location of recording sites.

### Freely moving optical stimulation and electrophysiological recording

The two-site multi-channel optrode system was composed of a micro-electrode array (eight tetrodes) and an optrode (optic fibre surrounded by eight tetrodes) that can be driven independently. Detailed information regarding the construction of the two-site device can be found in the Supplementary Methods. During surgery, two small craniotomies were performed at the location of the LA and ILSCm (or LP). Seventy-two hours after the surgery, neural activity monitoring was commenced. When stable spontaneous spikes appeared at both sites, the data recording was started and light stimulation in the ILSCm (or ILSCm–LP terminals) was delivered during periods when the animal was freely exploring. At the end of the experiment, recording sites were marked through electrolytic lesions for further histological verification.

### Single-unit spike sorting and analysis

Continuous wide-band data or discrete spike data were imported into Plexon Offline Sorter software for spike detection and offline sorting. To identify ChR2-expressing neurons, the characteristics of the neuronal responses to blue light were evaluated using three-parameters: (1) latency (<4 ms), (2) firing probability (>90%) and (3) cross correlation between the spontaneous and light-activated spikes (>0.9). To assess the response latencies of the LA neurons to upstream stimulation, the PSTH in the 40 ms (1 ms bins) following the light pulses was calculated.

### Trans-synaptic tracer labelling

All procedures were performed in BSL II animal facilities at the WIPM. To identify the relay station between the SC and LA, we combine the anterograde AAV labelling from the SC with rabies-mediated retrograde and trans-monosynaptic tracing from the LA. The retrogradely rabies virus system were used together with some helper AAV virus, and the advantages of this strategy are explained in the Supplementary Methods. First, 250 nl rAAV–CamkIIa–EYFP was injected into the SC with the following coordinate: (AP: −3.80 mm; ML: 0.50 mm; DV: −1.70 mm), and the mixed helper viruses containing AAV–CAG–GFP–ires–CRE, AAV–EF1a–FLEX–GT and AAV–EF1a–DiO–RV–G, (volume ratio: 1:2:3, total volume of 500 nl) were injected into the LA with the following coordinate: (AP: −1.50 mm; ML: 3.10 mm; DV: −4.60 mm). Two weeks later, 400 nl EnvA–RV–mCherry was injected into the LA at the same coordinates. Mice were sacrificed 1 week after rabies infection.

To retrogradely trace the afferent neural circuit from the LA, PRV-152 stocks mixed with CTB Alexa 594 (Molecular Probes, 0.05% in dH20) and calibrated in 1 × 10^9^ infecting unit per millilitre with 0.9% saline. A volume of 300 nl of the mixed solution was stereotaxic microinjected into the LA region. Because PRV can replicate and trans-multiple synapses in a time-dependent manner, mice were sacrificed at various stages of infection (36, 48 and 60 h, *n*=3 animals for each infection stage). For lesion studies, ibotenic acid (1% in dH2O, 300 nl) was injected ipsilaterally into the LP 9 days before the injection of PRV/CTB into the LA (*n*=3, mice were sacrificed at 65 h post infection). An equal volume of 0.9% saline was injected ipsilaterally into LP as control (*n*=4, mice were sacrificed at 60 h (*n*=1) or 65 h (*n*=3) post infection). The stereotaxic coordinate of LP is: (AP: −2.50 mm; ML: 1.50 mm; DV:−2.40 mm).Detailed information on the construction of the virus tracing system is provided in the Supplementary Methods.

### Immunohistochemistry and cell quantification

Mice were deeply anaesthetized and perfused transcardially with PBS, followed by ice-cold 4% paraformaldehyde. Brains were removed carefully and post fixed in PBS containing 4% paraformaldehyde at 4 °C overnight and cryoprotected in 30% sucrose solution for 3 days. Coronal brains slices (40-μm thick) were sectioned and the antibodies used were as follows: rabbit monoclonal anti-c-fos (dilution 1:200, 2,250; Cell Signaling), mouse monoclonal anti-VGLUT2 (dilution 1:200, MAB5504; Millipore), mouse monoclonal anti-PV (dilution 1:1m000, MAB1572; Millipore), mouse monoclonal anti-GAD67 (dilution 1:100, MAB5406; Millipore); rabbit anti-CamKIIa (dilution 1:250, AB52476; Abcam), and rabbit polyclonal anti-GFP (dilution 1:1,000, AB290; Abcam). After washing with PBS, the sections were incubated in the secondary antiserum Alexa Fluor 488 or 594 goat anti-mouse (dilution 1:100, C22842, Jackson) and anti-rabbit IgG (dilution 1:100, 711-607-003, Jackson). The sections were mounted onto microscope slides and covered with coverslips along with an anti-fade reagent containing DAPI (P36931, Invitrogen). The sections were then photographed and analysed using a Leica TCS SP5 laser scanning confocal microscope. Confocal images were acquired and cell counting was performed using image pro plus (Media Cybernetics, USA) or ImageJ software.

### Statistical analyses

One-way or two-way ANOVA, Kruskal–Wallis one-way ANOVA (an extension of rank test to three or more groups), student's *t*-tests and Mann–Whitney *U*-tests were used to determine statistical differences. *Post-hoc* analysis was applied to test individual differences between subgroups. Statistical analysis was conducted using SigmaPlot (Systat Software, California, USA). Graphs were made using SigmaPlot or Matlab (Mathworks, Natick, USA).

## Author contributions

P.W., N.L. and Z. Zhang contributed equally to this work. P.W., N.L., L.C. and L.W. designed the project and N.L. and P.W. initiated the project, performed virus or drug injections, optogenetic behaviour, electrophysiology experiments, collected and analysed the data. B.W., Y.L. and Z.Z. helped to collect the data. P.W. developed the signal processing toolbox. X.L., Y.L., Y.Z. and Y.T. performed immunohistochemistry and quantitative analysed the imaging data. Y.T. designed the micro drive optrode system and performed freely moving recording. X.H generated rabies and pseudo-rabies virus. X.H and Z. Zhang preformed virus injections and immunohistochemistry, and F.X. supervised, retrograde virus labelling experiments. L.X., L.C., G.B., X.H., F.X. and L.W. interpreted the results and commented on the manuscript. P.W. and L.W. wrote the manuscript. L.W. supervised all aspects of the project.

## Additional information

**How to cite this article:** Wei, P. *et al.* Processing of visually evoked innate fear by a non-canonical thalamic pathway. *Nat. Commun.* 6:6756 doi: 10.1038/ncomms7756 (2015).

## Supplementary Material

Supplementary Figures, Supplementary Methods and Supplementary References Supplementary Figures 1-11, Supplementary Methods and Supplementary References

Supplementary Movie 1 Acute silencing of CaMKIIa (+) ILSCm neurons in a mouse blocks the innate defensive responses to upper field *LS*. Mouse is stimulated with the upper field *LS* alone or in combination with optogenetics inhibition of CaMKIIa (+) ILSCm neurons (4X playing speed). The duration of *LS* is indicated by the appearance of "Stimulus on" text, and the duration of continuous 588-nm laser is indicated by the appearance of yellow "Light on" text.

Supplementary Movie 2 Optogentics *US* of CaMKIIa (+) ILSCm neurons in a mouse elicits exaggerated innate freezing followed by sustained anxiety-like responses and this response is quickly adapted by repeated application of stimulation. ChR2:ILSCm mouse is stimulated with the optogenetics *US* on the first and the fifth trial (4X playing speed). The duration of pulsed 473-nm laser is indicated by the appearance of *blue* "Light on" text. The duration of the freezing state is marked by the appearance of a *red* square at the *top right* corner.

Supplementary Movie 3 Optogenetics stimulation of DLSC-PAG pathway elicits backward fleeing. ChR2:DLSC-PAG mouse is stimulated with the 20-Hz pulsed 473-nm laser for 3 minutes (4X playing speed). The duration of pulsed laser is indicated by the appearance of *blue* "Light on" text.

Supplementary Movie 4 Reversible inactivation of the amygdala of a mouse blocks ILSCm *US*-elicited freezing response. ChR2:ILSCm mouse is stimulated with the optogenetics *US* at 20 min or 24 hr after muscimol infusion into the BLA.

Supplementary Movie 5 LP is a mono-synaptically relayed between ILSCm and LA. A reconstructed 3D video shows that the LP(LR) is expressed the ILSCm projecting terminals (*green*), which are adjacent to the retrograde trans-monosynaptic tracing labeled LP-LA cell bodies (*red*).

Supplementary Movie 6 Optogentics *US* of glutamatergic projection of ILSCm-LP(LR) elicits freezing. ChR2:ILSCm-LP(LR) mice were stimulated with the optogenetics *US* 20 min after glutamate receptor antagonist infusion into the LP.
