# Corrigendum: Processing of visually evoked innate fear by a non-canonical thalamic pathway

**DOI:** 10.1038/ncomms9228

**Published:** 2015-08-21

**Authors:** Pengfei Wei, Nan Liu, Zhijian Zhang, Xuemei Liu, Yongqiang Tang, Xiaobin He, Bifeng Wu, Zheng Zhou, Yaohan Liu, Juan Li, Yi Zhang, Xuanyi Zhou, Lin Xu, Lin Chen, Guoqiang Bi, Xintian Hu, Fuqiang Xu, Liping Wang

Nature Communications 6: Article number: 6756 (2015); 10.1038/ncomms7756 Published: 04092015; Updated: 08212015.

The affiliation details for Lin Xu and Xintian Hu are incorrect in this article. The correct affiliation for these authors is given below. Kunming Institute of Zoology, CAS Center for Excellence in Brain Science, Chinese Academy of Sciences, Kunming 650223, China.

