# Supplementary material for: Processing of visually evoked innate fear by a non-canonical thalamic pathway
Source: Nat Commun. 2015 Apr 9;6:6756. doi: 10.1038/ncomms7756 (PMC4403372; doi:10.1038/ncomms7756)
Supplement: Supplementary Figures, Supplementary Methods and Supplementary References — Supplementary Figures 1-11, Supplementary Methods and Supplementary References [file ncomms7756-s1.pdf]

Supplementary Figure 1

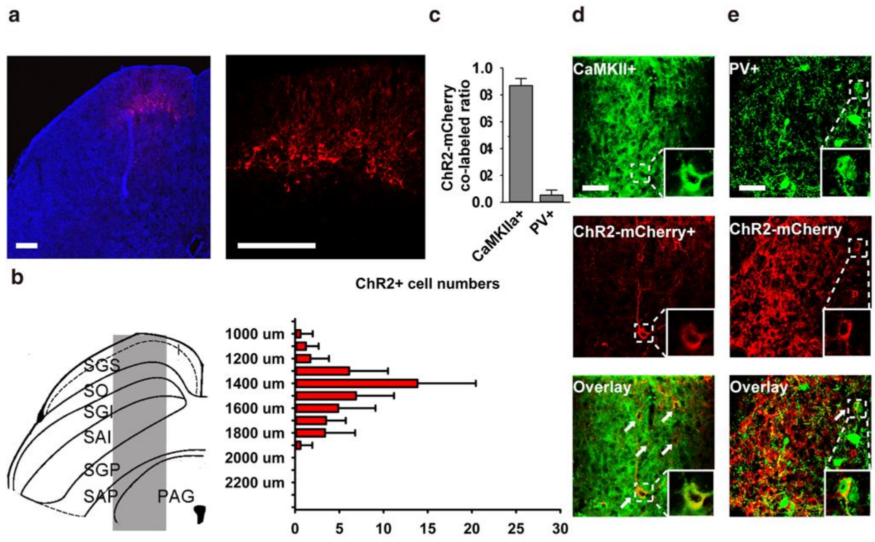

Supplementary Figure 1. ChR2 expression in the SC neurons. (**a**) A Confocal image of ChR2 expression in the SC (*red* = mCherry; *blue* =DAPI). *Right* panel shows that ChR2+ somata are mainly located at the surface of intermediate gray layer, and only sparse axon fibers are seen in the deep layers or PAG. (**b**) Distribution of ChR2+ cells within different SC layers (18 slices were harvested from 3 mice transfected with CaMKIIa-ChR2). *Left* panel indicates the cell-counting region (gray rectangle), middle panel shows the mean ± s.d number of neurons counted at the *AP* levels. (**c**) Cell-type specificity (CaMKIIa (+) and PV (+)) of ChR2-mCherry expressing cells. Note that about 5 % of CaMKIIa-ChR2:ILSC cells are co-labeled with PV. (**d**) CaMKIIa cells (*green*) stained with mCherry (*red*), examples of co-labeled neurons are indicated by *white* arrows in overlay image. Typical CaMKIIa+ neuron morphology is shown in the *bottom right*. (**e**) PV cells (*green*) stained with mCherry (*red*), examples of co-labeled neurons are indicated by *white*

arrows in overlay image. Typical PV+ neurons morphology is zoomed in the *bottom right*. Scale bars: **a**, 250 µm; **d**, **e**, 50 µm.

Supplementary Figure 2

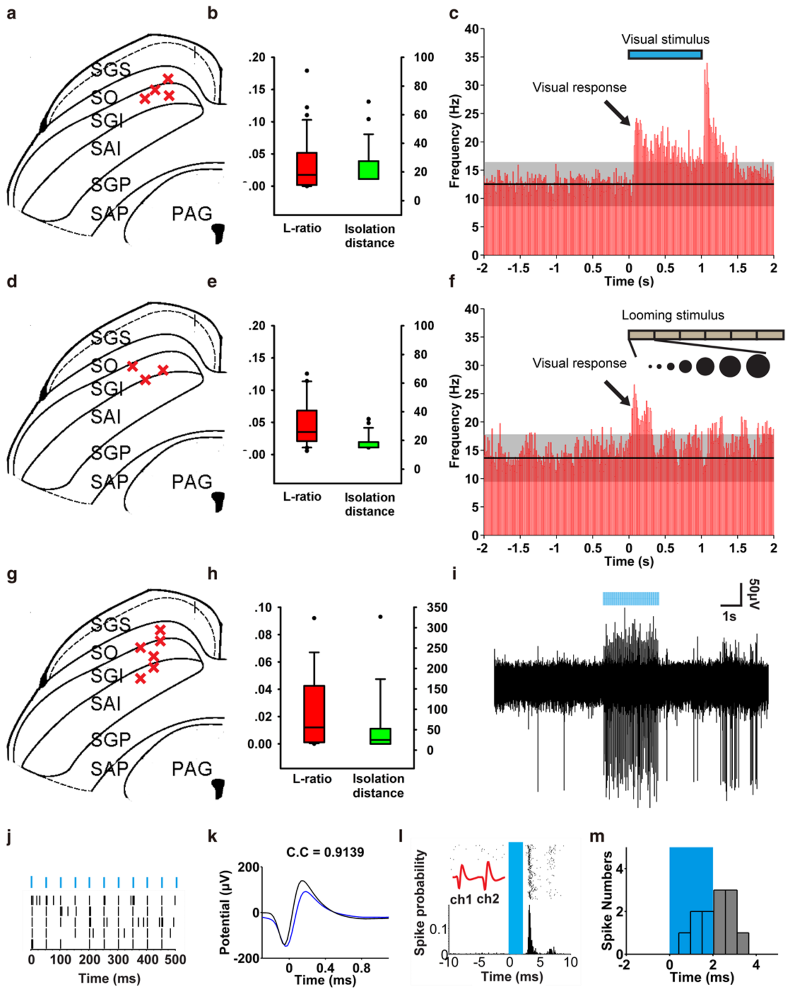

Supplementary Figure 2. Natural visual responses of ILSCm neurons and optical responses of ChR2:ILSCm neurons. (**a**, **b**) Natural visual stimulation was performed in four mice (1-s continuous *blue* light for at least 15 trials at 10 s intervals) and a bundle of tetrodes were implanted in the ILSCm; 36 units were recorded and well isolated from 8 experiments (note that because the tetrodes are drivable, neural activities at different sites can be recorded from one animal), the tips of the tetrodes at the last recording site are shown in (**a**)**.** To measure the quality of cluster isolation, two measurement criterions on sorted spike units were used: L-ratio

and isolation distance. A well isolated single unit followed the rules of L-ratio < 0.2 and isolation distance > 15, (**b**) shows these 36 units have the averaged single unit L-ratio = 0.03±0.04 and isolation distance = 24±19. (**c**) The population firing of ILSCm neurons shows transient peak during 60 ~ 170 ms (10- ms bins) following stimulus onset, the averaged firing rate in this short-latency visual response period was 21 Hz. (**d**, **e**) Looming stimulation was performed in three mice (a looming trial consists of 15 looming stimulus blocks with a total duration of 5 seconds; the looming trial is repeated at least 15 times in an experiment) and a bundle of tetrodes were implanted in the ILSCm; 24 units were recorded and well isolated from 5 experiments, (**d**) shows tips of tetrodes, (**e**) shows these 24 units have the averaged single unit L-ratio = 0.05±0.03 and isolation distance = 18±5. (***F***) The population firing of ILSCm neurons shows a transient peak between 40 ~ 140 ms (10- ms bins) following the first looming stimulus block onset. The averaged firing rate during this period was 23 Hz. (**g**, **h**) Based on the magnitude of the population visual response of ILSCm neurons, a 20-Hz stimulation frequency was chosen for optogenetics modulation. Three ChR2:ILScm mice were anesthetized and optical stimulation and recording was performed. A total of 12/17 units were identified as the ChR2+ neurons. (**g**) Shows the placement of the optrode (the depth of the electrode location is read from the reading on the stepping motor drive), (**h**) shows the recorded 17 units have the averaged single unit L-ratio = 0.03±0.02 and isolation distance = 40±15. (**i**) An example of an ILSCm neuron that was activated by optical stimulation. (**j**) Raster plots of the spikes in different trials for the neuron in (**i**), showing that the firing time

(*black* bars) is followed by the stimulating light pulses (*blue* rectangle). (**k**) For a well-light-responsive ChR2+ ILSCm neuron, the correlation coefficients between the waveforms of light-evoked (*blue*) and the spontaneous spikes (blank) were > 0.9. (**l**) Peri-stimulus time histogram and raster (*PSTH*) of the neuron in (**i**) is aligned with the light pulse (*blue* rectangle) onset and the corresponding spike waveforms are shown in the inserted panel (*red*). (**m**) Histogram of spike latency to light pulses onset for 12 identified ChR2+ neurons.

Supplementary Figure 3

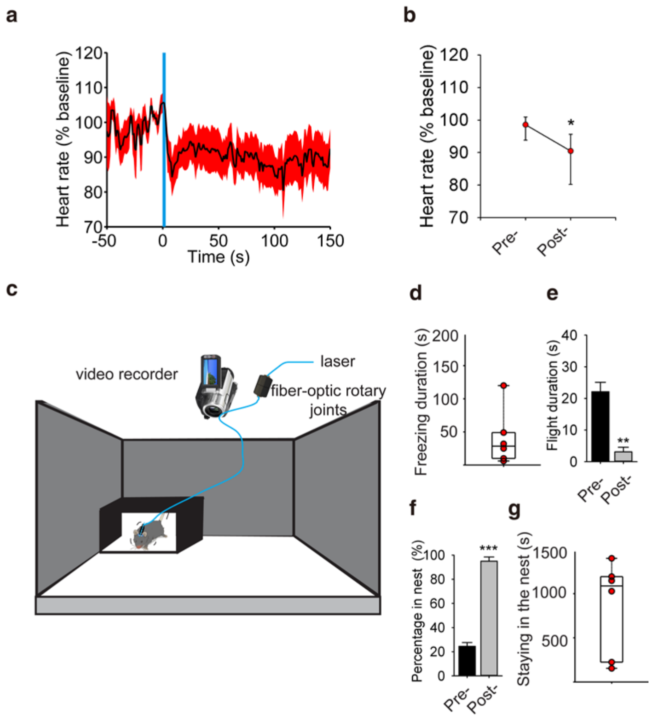

Supplementary Fig. 3. Optogenetic activation of ILSCm induced bradycardia activity and nest escaping behavior after *UF*. (**a**) Time courses of averaged HR against to baseline revealed that ChR2:ILSCm (*red*) *US* (*blue* rectangle) elicited sustained bradycardia activity. (**b**) Averaged HR reduced about 10% after ChR2:ILSCm optical stimulation. (**c**) Schematic of the testing environment with a protective nest. (**d**) Box plots of *UF* durations elicited by *US*. (**e**) Averaged interval between animal leaved and back to the test. (***F***) Averaged time percentage of animal stayed in the nest for 5 minutes. (**g**) Averaged interval between animal escaped to nest and out of nest again.

Supplementary Figure 4

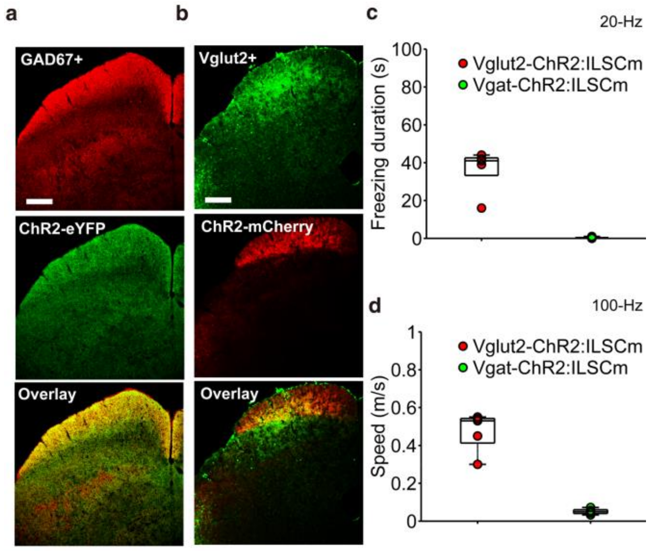

Supplementary Fig. 4. Optogenetic activation of Vglut2-ChR2:ILSCm and Vgat-ChR2:ILSCm. (**a**) Confocal images of ChR2 expression in the GABAergic SC neurons of Vgat-ChR2-EYFP mice (*red* = GAD67; *green* = EYFP). (**b**) Confocal images of Cre-dependent ChR2 virus infection in the glutamatergic SC neurons of Vglut2-ires-cre mice (*green* = Vglut2; *red* = mCherry). (**c**) Only 20-Hz optical *US* of Vglut2-ChR2:ILSCm elicited *UF* rather than Vgat-ChR2:ILSCm. (**d**) 100-Hz high frequency optical stimulation of Vglut2-ChR2:ILSCm elicited animal flight and wild-running behaviors, which was reflected by an extremely high moving speed during the laser light “on” period (almost 10-fold more than normal speed). Vgat-ChR2:ILSCm mice didn’t show these responses. Scale bars: **a**, **b**, 250 µm.

Supplementary Figure 5

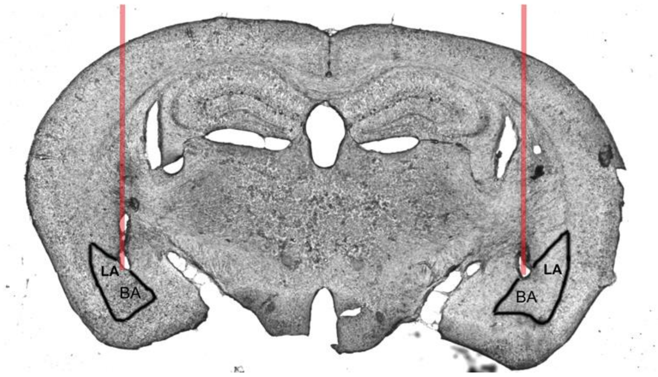

Supplementary Fig. 5. Locations of muscimol:BLA injections. Coronal section shows that the muscimol was injected in the bilateral BLA, *red* bars indicate the placement of cannulas.

Supplementary Figure 6

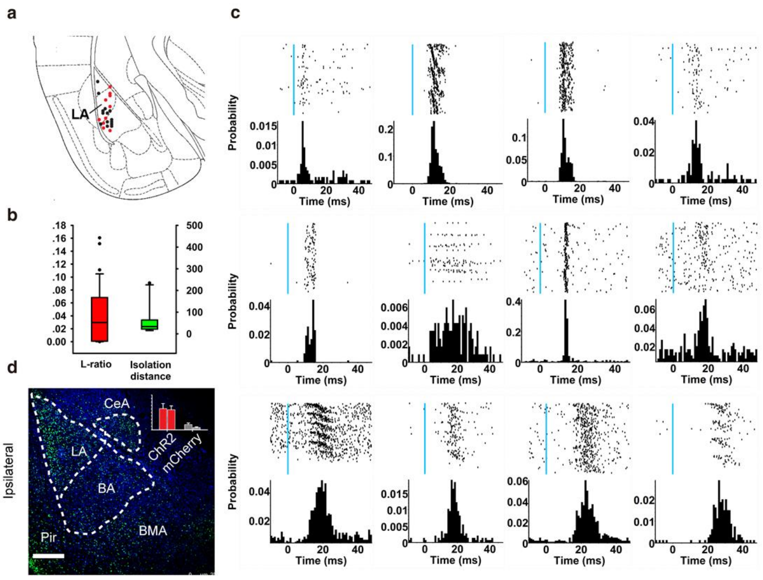

Supplementary Fig. 6. The ILSCm stimulation and the LA recording in anesthetized mice. (**a**) Recording sites in the LA. Twenty recording sites from 7 mice are shown. Recorded location of LA responsive cells is marked with *red* dots and the *black* dots indicate no responses. (**b**) 38 LA single units were well isolated with averaged L-ratio = 0.04±0.04 and isolation distance = 92.3±212.5. (**c**) Z-scored peri-stimulus time histograms and rasters (*PSTH*) aligned with onset of light pulse in ILSCm for 12 responsive cells in LA. (**d**) ChR2:ILSCm stimulation resulted a large number of c-fos+ expressing cells in the LA in anesthetized animals with no evoked behavioral responses. The inserted histogram shows the comparison of c-fos+ cells in the bilateral LA between the ChR2 and mCherry groups ($P < 0.001$). (*green* = c-fos; *blue* = DAPI; *left* bar = ipsilateral side; *right* bar = contralateral side). Scale bars: **d**, 250 μm.

Supplementary Figure 7

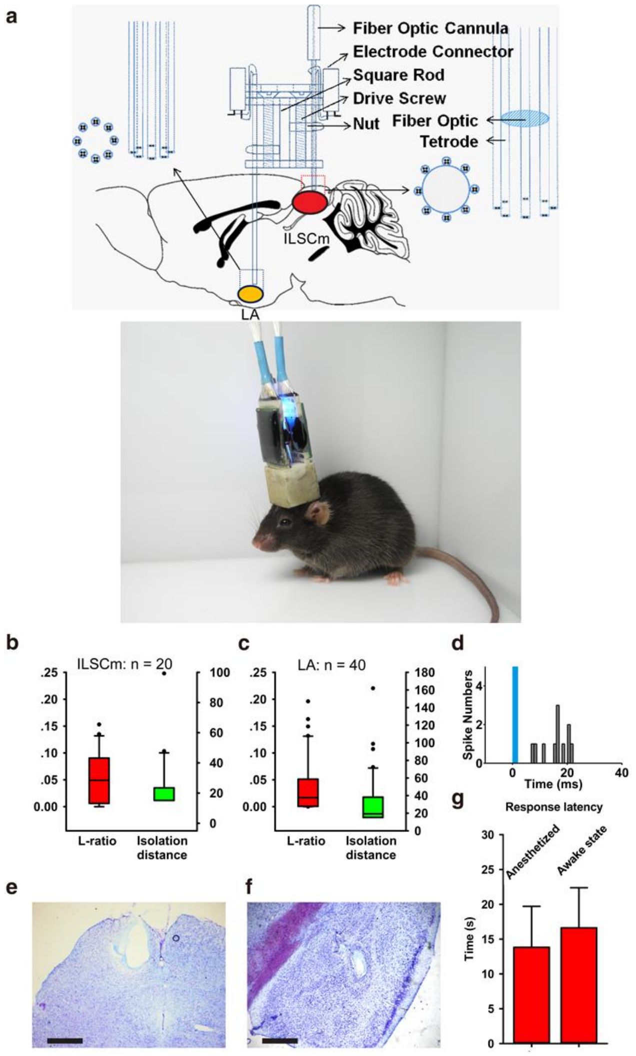

Supplementary Fig. 7. Stimulation/recording in the ILSCm and simultaneous recording in the LA of freely moving mice. (**a**) *Top*, schematic of the construction of the two-site multi-channel optrode system: a bundle of 8 tetrodes (32 channels) and an optrode (optic fiber attached with 8 tetrodes around) were setup on the microdrive system, and the placements of the two bundle holes were pre-arranged on the custom board by calculating the relative position of the targeted areas in the horizontal plane. (the ILSCm or LA here). *Bottom*, the recording system was mounted on a

ChR2:ILSCm mouse and a 473nm laser was delivered through the fiber optic. (**b**, **c**), Single units can be readily separated in the ILSCm (**b**, 20 neurons with L-ratio = 0.05±0.06 and isolation distance = 22.1±16.5) and the LA (**c**, 40 neurons L-ratio = 0.03±0.04 and isolation distance = 31.2±28.3). (**d**) Histogram of peak response latencies to light pulses onset (*blue* rectangle) for 11 identified LA responsive cells in freely moving mice. (**e**, ***F***), Nissl staining reveals the locations of the optrode in the ILSCm (**e**) and the tetrode in the LA (***F***). (**g**) The latencies of LA responsive cells under freely moving and anesthetized recording conditions were not significantly different ($P > 0.05$). Scale bars: **e**, ***F***, 500 μm.

Supplementary Figure 8

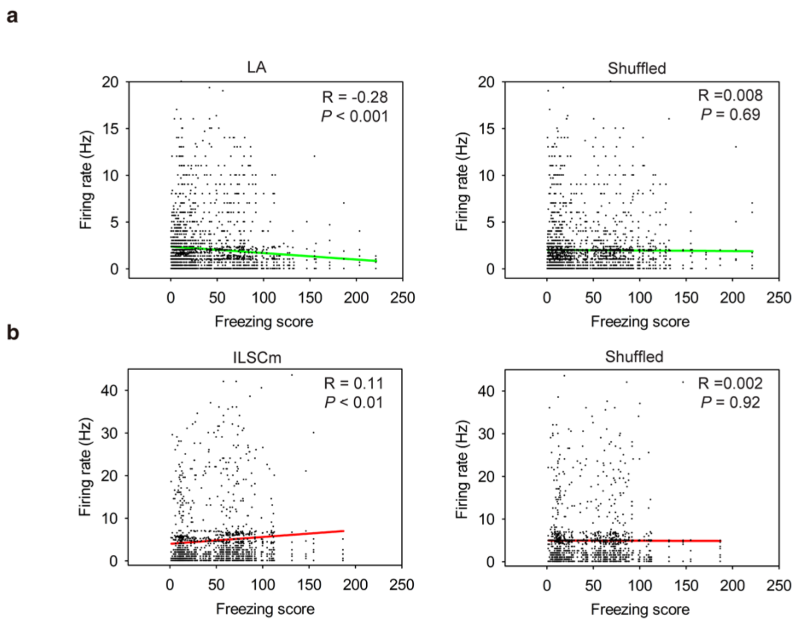

Supplementary Fig. 8. Correlation between the neural activities and freezing score. (**a**) *Left* panel: firing rate (1-s bin) in the LA plotted against the freezing score, the selected time window was from pre-stimulation 50 s to post-stimulation 150 s. *Green* line represents the linear fit curve. The result showed the firing rate of the LA neurons negatively correlated with the freezing score, which means LA neurons have more strong activation during animal freeze. *Right* panel: control (shuffled data). (**b**) As in **a** for data from ILSCm. This result indicates the ILSCm neurons show silencing during animal freeze.

Supplementary Figure 9

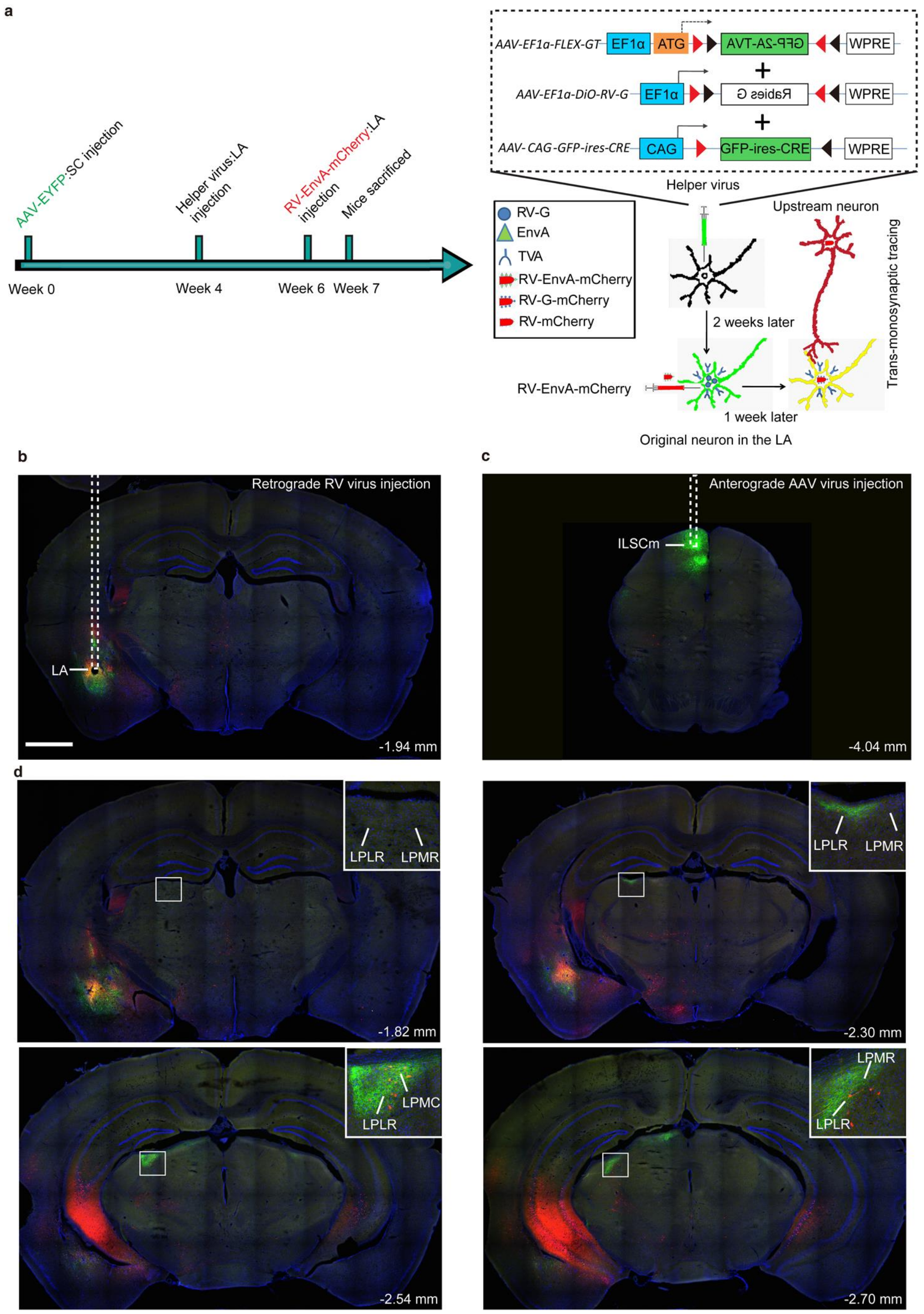

Supplementary Fig. 9. Combining anterograde labeling with retrograde and trans- monosynaptic labeling to trace the SC-LP-LA circuit. (**a**) The time line of

the strategy for combining the anterograde AAV-EYFP:ILSCm terminals labeled with retrograde and trance-mono-synaptic RV-Cherry:LA tracing. (**b**) The sketch map shows the strategy of retrograde and trans-monosynaptic tracing from the LA projection neuron. The expression of Camkiia-driven Cre elicited the specific expression of GFP-2A-TVA. as well as RabiesG. (**c**, **d**), Coronal brain sections show the injection positions of RV-mCherry:LA in combination with helper AAVs (**C**) and the bilateral AAV-EYFP:ILSCm (**d**). (**e**) The co-localization of the anterograde AAV-EYFP:ILSCm terminals (*green*) and retrograde RV-mCherry:LA cells (*red*) in the LP subdivisions. Scale bars: **b-d**, 1 mm.

Supplementary Figure 10

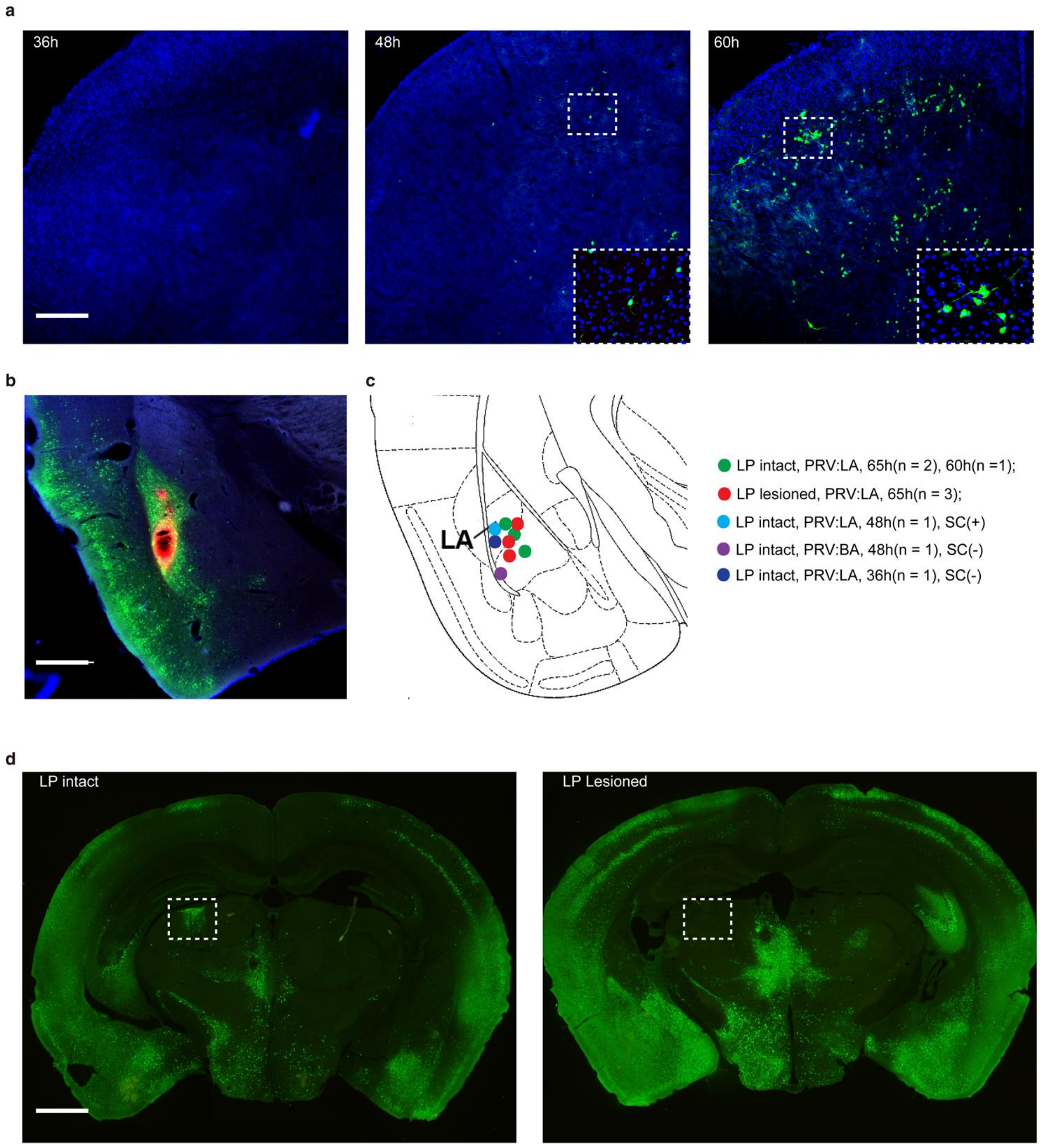

Supplementary Fig. 10. PRV152-EGFP mediated retrograde and trans-multiple synaptic tracing from LA. (**a**) Confocal images of PRV-EYFP:LA labeled cells in the SC at increasing post-injection times. *White* boxes in the inserted panel depict the areas (*green* = PRV; *blue* = DAPI). (**b**) The location of the PRV injection is indicated by the cholera toxin subunit b (CTb)-594 fluorescence staining, which was injected at the same time as the viruses (*red*). (**c**) Schematic plots of the tips of PRV injection

sites in all cases. Unlike injections in the LA where PRV+ cells in the SC can be observed 48 hours post-injection (middle panel in **a**), PRV injected in the BA does not label any cell in the SC at the same time point (data not shown), suggesting that BA to SC might be two synapses away. (**d**) Comparison between the two coronal brain sections shows the PRV (+) cells are no longer expressed in the LP that has the lesion, although the labeling is stronger in the other brain regions. Scale bars: **a**, 250μm; **b**, 500μm; **d**, 1mm.

Supplementary Figure 11

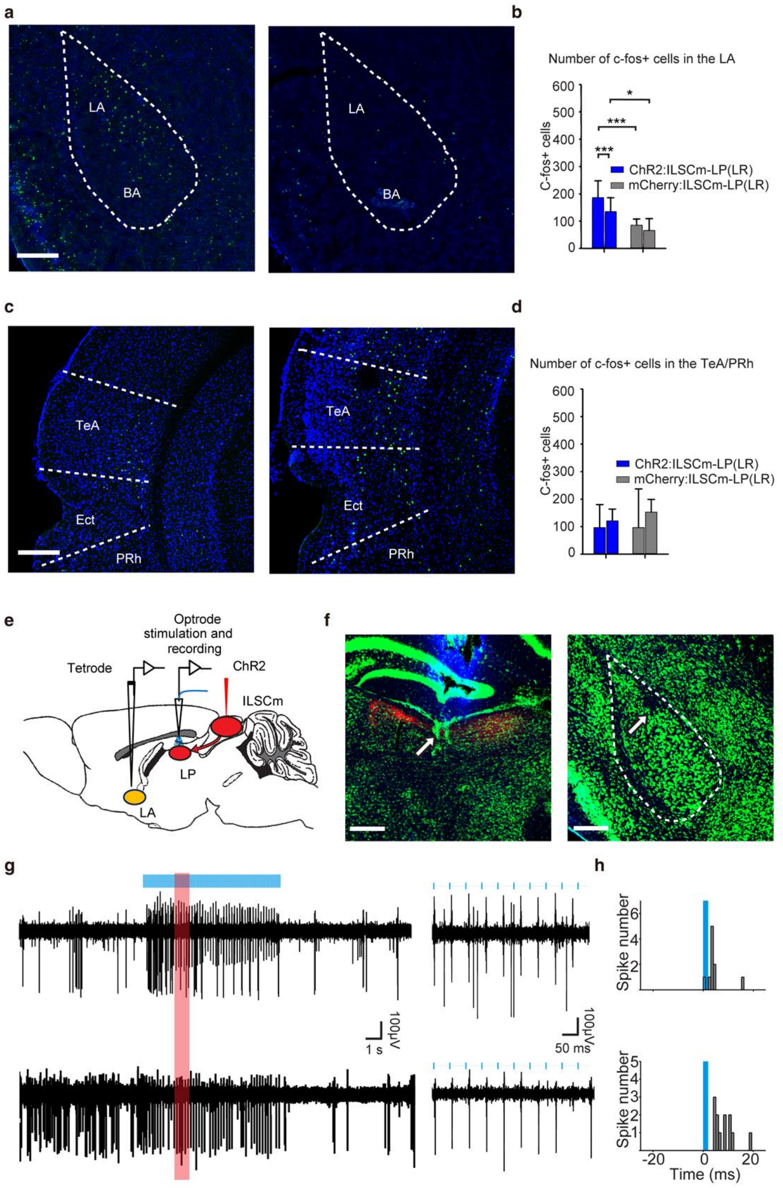

Supplementary Fig. 11. Optogenetic activation of ILSCm-LP projection elicits LA neuron responses. (**a**) Images of the amygdala, stained for c-fos, from a ChR2:ILSCm-LP(LR) or mCherry:ILSCm-LP(LR) mouse 30 min after *US* (*green* = c-fos; *blue* = DAPI). (**b**) Average c-fos (+) cell counts in the LA showed that the bilateral c-fos levels of ChR2: ILSCm-LP(LR) mice were higher than in mCherry:ILSCm-LP(LR) mice (*left* bar = ipsilateral side; *right* bar = contralateral

side) (*n* = 15 slices per group; ****P* < 0.001, **P* < 0.01, **P* < 0.05 by two-way ANOVA with Holm-Sidak post hoc test). (**c**) Images of the TeA and the PR, stained for c-fos, from a ChR2:ILSCm-LP(LR) mouse or mCherry:ILSCm-LP(LR) mouse 30 min after *US*. (**d**) Average c-fos (+) cell counts in the TeA and the PR showed no significant difference between two groups (*left* bar = ipsilateral side; *right* bar = contralateral side) (*n* = 15 slices per group; two-way ANOVA with Holm-Sidak post hoc test). (**e**) Schematic of optrode stimulation and recording in the ILSCm-LP and simultaneously tetrode recording in the LA. (***F***) Fluorescent Nissl staining reveals the tip of the optrode in the LP (*white* arrow). (*green* = Nissl; *red* = mCherry; *blue* = DAPI). (**g**) The tip of the tetrode in the LA (*white* arrow). (**h**) Example LP (*top*) and LA neurons (*bottom*) are shown that responded the 20-Hz optogenetics stimulation of ILSCm-LP(LR) terminals. (**i**) Histogram of peak response latencies to the onset of light pulses (*blue* rectangle) for 10 identified LP response neurons (*top*) and 13 identified LA response neurons. Values are mean ± s.d, Scale bars: **b**, 250 µm; **c**, 250 µm; **d**, 250 µm.

Supplementary Methods

Subject. Adult (25-30 g) male C57BL/6 mice (Guangdong Medical Laboratory Animal Center, Guangzhou, China), adult male Vgat-channelrhodopsin (ChR2(H134R))-EYFP mice (from Guoping Feng's lab, which transgenically express ChR2 in GABAergic neurons under control of Vgat2 (Slc32a1) promoter) and adult male Vglut2-ires-cre mice (from Jackson Laboratory, which express Cre recombinase from the endogenous Vglut2 (Slc17a6) promoter), aged 6-8 weeks at the beginning of experiments, were maintained with a 12 h/12 h light/dark cycle (lights on at 08:00 am , Beijing time) and given food and water ad libitum. Animal husbandry and all aspects of experimental manipulation were in accordance with the Institutional Animal Care and Usage Committee of Shenzhen Institutes of Advanced Technology (SIAT), Chinese Academy of Sciences (CAS).

Virus construction and packaging. AAV vectors encoding pAAV-CaMKIIα-third-generation halorhodopsin (eNpHR3.0)-EYFP (Virus A), pAAV-CaMKIIα-mutant version of ChR2 (hChR2 (E123T/T159C))-mCherry (Virus B)、pAAV-CaMKIIα-mCherry (Virus C) and pAAV-DIO-hChR2 (H134R)-mCherry (Virus D) were obtained from Karl Deisseroth's lab. High titer AAVs (>10^{12} viral particles/*ML*) were serotyped with AAV5 coat proteins and packaged using calcium phosphate precipitation methods. Briefly, AAV vectors were co-transfected with pHelper and pRC5 into HEK293 cells. 72 hours later, cells were collected, lysed and loaded onto 8 % PEG8000/0.6 M NaCl solution for centrifugation at 3000 g for 30

minutes. The resulting viral pellet was resuspended in 100 μl of phosphate buffered saline.

Local virus and drug injection and optic fiber implantation. All surgeries were performed under aseptic conditions under stereotaxic guidance [1]. Mice were anaesthetized with pentobarbital sodium (0.5 % W/V, 80 mg/kg body weight, i.*P*.) (Merck, Darmstadt, Germany), then placed in a stereotaxic apparatus (RWD, Shenzhen, China). Erythromycin eye ointment was applied to prevent corneal drying and a heating pad (RWD, Shenzhen, China) was used to hold body temperature to 37 ℃. A small incision was made to expose the skull surface and to visualize bregma and lambda. 0.7 μl AAV (Virus A, B or C) was injected (with rate of 100 nl/min) from a 10 μl microsyringe with a 33 gauge metal needle (Neuros; Hamilton, Reno, USA) connected to a microsyringe pump (UMP3; WPI, Sarasota, USA) and its controller (Micro4; WPI, Sarasota, USA) into the bilateral intermediate layers of the superior colliculs, medial part (ILSCm) (*AP*: -3.70mm; *ML*: ±0.60mm; *DV*: -1.85 mm) (Virus A, C), unilateral ILSCm (Virus B, C) or intermediate layers of the superior colliculs, lateral part (ILSCl) (*AP*: -3.70mm; *ML*:1.30mm; *DV*: -2.20 mm) (Virus B) of C57BL/6 mice, Virus D was injected in the unilateral ILSCm of Vglut2-ires-cre mice. After the injection finished, the needle was lifted 100 μm and *left* in the place for an extra 10 minutes to facilitate the diffusion of the virus and then withdraw slowly.

For the SC somata optogenetics stimulation only, a home-made implantable optic fiber [2] (NA = 0.37, *Φ*=200 μm; Fiblaser, Shanghai, China) was inserted into the

ILSCm (*AP*: -3.70mm; *ML*: 0.50 mm; *DV*: -1.40 mm), or the ILSCl (*AP*: -3.70mm; *ML*: 1.25 mm; *DV*: -1.80 mm). For the ILSCm-lateral posterior nucleus of the thalamus (LP) terminals stimulation, the fiber was implanted at (*AP*: -2.40mm; *ML*:1.50 mm; *DV*: -2.10 mm) to target ChR2:ILSCm projecting terminals in the LP. For yellow light stimulation of dual ILSCm, the the optic fiber was implanted at a 20° from the vertical axis at the following coordinates (*AP*: -3.70mm; *ML*: ±1.25 ; *DV*:-1.50 mm). While for both optogenetics stimulation and drug apply, the optic fiber was implanted at a 15° from the vertical axis (*AP*: -4.10mm; *ML*: 0.50 ; *DV*: -1.45 mm). Guide cannulae (OD = 0.48 mm, with dummy screw caps, RWD, Shenzhen, China) were implanted bilaterally to inject in BLA at the following coordinates (*AP*: -1.70 mm; *ML*: ±3.20 mm; *DV*:-3.60~3.70 mm), or in unilateral LP (*AP*: -2.40mm; *ML*: 1.50 mm; *DV*: -1.60 mm). The mating injectors were just 1 mm longer than the tips of cannulae and the fibers were about 0.5 mm longer than them. Stainless steel stylets with dummy caps were inserted into cannulae to maintain patency.

Implants were fixed to the skull with at first a layer of cyanoacrylate glue (Permabond, Englewood, USA) and flowed by dental cement (New Century, Shanghai, China). After the surgery, the skin was sutured and lincomycin hydrochloride and lidocaine hydrochloride gel was applied to prevent inflammation and decrease pain. Electrophysiological and behavioral experiments were performed after at least 4 weeks to allow for viral expression. For terminals stimulation, the duration of viral incubation last for an extra 2-4 weeks.

***In vivo* anesthetized stimulation and recording**. Mice were anesthetized with Urethane (10 % W/V, 1.9g/kg body weight, i.*P*.) (Sigma, USA) and secured in the stereotaxic apparatus. Skin above the skull was removed and small holes were drilled with a dental drill (RWD, Shenzhen, China). A 0.37 *N*.A. 200 μm core diameter optic fiber was placed in the ILSCm (*AP*: -4.1 mm; *ML*: 0.5 mm; *DV*: -1.45 mm at a 15° from the vertical axis) and connected to a DPSS *blue* light laser (MBL-473/200 mW; Fiblaser, Shanghai, China). The power of the *blue* light was set to about 7-10 mW at the tip of the fiber using a power meter (Thorlabs, Newton, USA) and the laser pulses were modified by an arbitrary/function generator (AFG3000B; Tektronix, Beaverton, USA). A micro-electrode array (MEA) was inserted in the LA with the help of a stepping motor (IVM; Sentifica, Uckfield, USA) at a speed of 1 μm/s (*AP*: -1.7 mm; *ML*: 3.2 mm; *DV*: 3.5-4.5 mm below the dura). The array consisted of 8 stereotrodes made by insulated nichrome wires (OD = 17 um; CFW, California, USA), which were electrochemical modified until the impedance was less than 0.5MΩ. Data were collected by OmniPlex® D Neural Data Acquisition System (Plexon, Dallas, USA). 20-Hz with 2 ms width pulsed laser was delivered in the ILSCm when stable spontaneous spikes of the LA neurons were detected. At the end of the experiment, recording sites were marked through electrolytic lesions (0.1 mA DC, 20 s). Mice were sacrificed immediately and perfused transcardially. For histological verification of the recording site, 60-μm coronal brain sections were made on a cryostat microtome (CM1950; Lecia, Wetzlar, Germany) and imaged on a microscope (FN1; Nikon, Tokyo, Japan).

In vivo* freely moving stimulation and recording.** ***Construction of the two-site multi-channel optrode system. The two-site multi-channel optrode system was composed of a MEA (8 tetrodes) and an optrode (optic fiber attached with 8 tetrodes around), which can be driven independently. The fiber tip was 0.5 mm shorter than the tetrode tips. The input end of the fiber was embedded into a ceramic ferrule, which could be connected to the laser source via FC/PC connector. The tips of all tetrodes (12.7 μm tungsten wires, CFW, California, USA) were plated with platinum (chloroplatinic acid solution) to a final impedance of 0.5–0.8 MΩ. Two bundles were arranged in print circuit board appropriately according to the horizontal location of the ILSCm (or the LP) and the LA. The driver systems had a range of 5 mm advance-distance independently. Since the polyimide cannulae were glued to the driver nut with Epoxy (353ND, Precision Fiber Products, Milpitas, USA), the electrodes could advance 300 μm by turning the drive screw one circle. We then were able to lower the electrodes in dozens of micrometers step by step by controlling the rotation of the screw.

Surgery. Male C57BL/6 mice accepted virus injection were housed individually and handled 2 or 3 times a day until they are habituated to the operator. Mice were anesthetized with pentobarbital sodium (80 mg/kg body weight) and secured in the stereotaxic apparatus. Then remove the subcutaneous layers of tissues, expose the skull and deal with hydrogen peroxide to make the surface clearly. Two small craniotomies were made at following coordinates: *AP*:-2.1 mm; *ML*: 3.0 mm and *AP*:-3.7 mm; *ML*: 0.5 mm (or *AP*: -2.40mm; *ML*:1.50 mm), corresponding to the LA

and ILSCm (or LP) respectively. Four screws were fixed on the skull around the craniotomies, and packed with dental cement to make a firm steel frame. The two-site multi-channel optrode system was lowed through the drilled holes slowly until the two adjustable MEA were placed 1mm above ILSCm (or LP) and LA, then sealed the holes with softened paraffin and weld the reference to the wire linked to the screw insert in the skull. Last, the whole system was secured with dental cement linked to the steel frame mentioned above. At the end of the experiment, recording sites were marked through electrolytic lesions for further histological verification using fluorescent Nissl staining.

Multi-channel recording. 72 hours later after the surgery, electrophysiological activities of the animal were acquired by OmniPlex® D Neural Data Acquisition System. The micro-drive screw was turned 1/4 cycle one time to advance the electrode 70 μm deeper until the electrode tips reached the ILSCm (or LP) and the LA. Then the screw was turned a few times again at a rate of less than 1/16 cycle one time to try to detect the good signal-to-noise (SNR, generally > 3 was kept) spikes, thus this step may carry on a couple of days with patience. When the stable spontaneous spikes appeared in both two sites, we started to record the data and optogenetics *US* (20-Hz frequency with 2 ms pulse width for 2.5 s) was delivered in the ILSCm (or ILSCm-LP terminals) during the animal freely exploring.

Single-unit spikes sorting and analysis. Continuous wide-band data (anesthetized recording using the stereotrodes) or discrete spike data (freely moving recording using

the tetrodes) were imported into Plexon Offline Sorter software (Plexon Inc., Dallas, USA) for the spike detection and offline sorting. The multi-unit spikes in the continuous wide-band data were firstly detected as the events which exceeded a threshold of -4.5 sigma of root mean square (RMS) and the spike waveform length was set as 1.4 ms (56 bins). Then units cluster was isolated using the principal components in each electrode. An automatic T-Dist E-M clustering method was performed to sort spikes, and some minor adjustments were operated manually to exclude the obvious artifacts. A single units cluster should have a clear refractory period (>1 ms), thus only clusters containing < 1 % of the spikes with ISI < 1 ms were included. Next, the sorted waveforms and timestamps of all clusters were sent to the Matlab (Mathworks, Natick, USA) workspace. Before the further analysis, two additional procedures were performed to evaluate sorting quality and avoid the repeated analysis of a same neuron recorded on different channels. 1) Two quantitative metrics of sorting quality were used: L-ratio, which represents the degree of noise contamination of a cluster, and isolation distance, which is the estimation of Mahalanobis distance from a specific cluster center to other spikes [3]. The candidate clusters were selected as the well isolated single units following the rules of L-ratio < 0.2 and isolation distance > 15 [4]. 2) To avoid the possibility that more than one single units on different channels represented the same neuron activity, the cross-correlation parameter was measured: if the cross-correlation histogram reveals a significant sharp peak within in ±1ms, then only one of them was selected for the further analysis.

To identify ChR2 expressing neurons from all well-isolated single units, the characteristics of the neuronal responses to *blue* light (λ = 473 nm) stimulation was reflected by three-parameters: 1) latency: the *PSTH* in 10 ms (0.1-ms bins) following the light pulses during 20-Hz stimulation was calculated, the neuronal response latency was taken as the first bin in the light pulsed triggered *PSTH* that exceeded the 95 % confidence limits of the averaged firing rate in the 10-ms preceding the light pulses. If the response latency could not be elicited in 10 ms following the light pulses, this neuron was considered as the no-responses neuron; 2) probability: the neuronal firing probability was calculated from the integration of probability light pulsed triggered *PSTH*; 3) waveform correlation coefficients (C.C) between spontaneous and light-evoked spikes in a sorted unit. These parameters were constructed a feature space and sent to an unsupervised hierarchical clustering classifier, an cluster of neurons with short peak response latency (< 4 ms), high firing probability (> 90 %), and high C.C (> 0.9) were subtracted from the feature space and identified as the ChR2 expressing neurons that can be directly activated by light.

To assess the response latencies of the LA neurons to ILSCm somata or ILSCm-LP terminals light stimulation, the *PSTH* in 40 ms (1-ms bins) following the light pulses during 20-Hz stimulation was calculated and the latency detection procedure is same as mentioned above.

Behavior assay. ***Looming.*** Looming apparatus and the testing paradigm was mentioned in [5]. In brief, the apparatus was a 40 length × 40 cm width × 30 cm height

closed box, which consisted of a *white* frosted organic plastic floor and ceiling and 4 transparent plexiglass side walls. All the side walls were coated to be unidirectional perspective, so that the mouse couldn't see the outside but its movement can be recorded at 30 fps by a HD digital camera (Sony, Shanghai, China) from the outside of apparatus. A LCD monitor was embedded in the ceiling and kept its screen gray to supply basic illumination to the arena. Looming stimulus (*LS*) was programmed by E-prime software (Psychology Software Tools, Inc, Sharpsburg, PA, USA) and was displayed on the monitor. An *LS* was a vision of a *black* disc appeared directly above the animal at a diameter of 2 degrees of visual angle, expanded to 20 degrees, which was quickly repeated 15 times in 5 s.

All animals were handled at least 5 times in one week before the tests. During the testing day, mouse was first put in the apparatus and habituated itself to the box for at least 10min. In this study, the shelter nest was not placed in the apparatus and *LS* was triggered by the experimenter while the mouse entered the center zone of the arena. The second *LS* was repeated after about 3min. Each animal was put in a new cage after the test to prevent any interaction between tested and naïve mice and then return to the home cage when all the behavior tests finished.

For optogenetics inhibition experiment, two implanted optic fibers were connected to a yellow light laser (MBL-589/200 mW; Fiblaser, Shanghai, China) via custom patch cords (Fiblaser, Shanghai, China) and a fiber-optic rotary joint (FRJ_1*2i-FC-2FC; Doric Lenses, Quebec, Canada) which could release the torsion

of the fibers result from rotation of the mouse. The power of the yellow light was set to about 15-20 mW at the fiber tips. The optical stimulation was delivered into the SC while the first *LS* onset, and the duration of continuous optical stimulation fully covered that of the *LS*. The second *LS* was repeated after about 3min while the yellow laser was shut downed.

Optogenetics stimulation. Experiments took place in a rearing cage (40 cm length × 30 cm width × 20 cm height) which was carpeted with litter. All animals were handled at least 5 times in one week before the tests. In addition, heart rate monitoring group were shaved around the neck and habituated to a neck collar sensor for three days prior to the tests (MouseOx Plus; Starr Life Sciences, Allison Park, PA, USA), and drug injection experiments group of mice were habituated to dummy caps removal and replacement. For optogenetics manipulations, the implanted optic fiber was connected to a *blue* light laser via patch cords and a fiber-optic rotary joint (FRJ_FC-FC; Doric Lenses, Quebec, Canada). The power of the *blue* light was set to about 7-10 mW at the fiber tip for cell body stimulation and 15-20 mW for terminals stimulation. The laser pulses were modified by an arbitrary/function generator.

On the first day (day 1), the mouse with fiber patch cord connected was put in the cage to acclimatize to the apparatus for a minimum of 30 min. On the next day (day 2), the test commenced with a 5 minutes freely exploration period as the baseline, and then 5 repeated *blue* light *US* trials (50 laser pulses of 2 ms at 20-Hz) were delivered in ILSCm somata or ILSCm-LP(LR) terminals. The inter-trial interval was set about 3

minutes which was controlled manually, the moments for triggering on the light were always chosen at period of mouse freely moving. After all the light stimulation finished, another 5 min period was recorded as a laser off response.

Drugs administration. On the first day (day 1), mice were also habituated to the apparatus as described above. On the next day (day 2), for the experiment of GABAA receptor agonist injection in the BLA, the dummy caps were removed first, and then muscimol (Sigma-Aldrich, Shanghai, China) in 0.2μl phosphate buffered solution (PBS) was infused at 0.1μg or 0 (the control group) per hemisphere at a rate of 0.1 μl/min through two stainless steel injectors (OD = 300 μm; RWD, Shenzhen, China) into bilateral BLA via the implanted cannulae. The injectors were attached with PEBAX tubing to the respective 10 μl Hamilton syringe controlled by a microsyringe pump. After injection completed, the injectors were *left* in place for an extra 2 minutes to facilitate the diffusion of the drug. Then pulled them out and reinserted the dummy caps. 20 minutes after the drug injection completed, the *US* of ChR2:ILSCm or ChR2:ILSCm-LP(LR) tests were performed as mentioned above, but mice received only one trial laser stimulus. 24 hours later (day 3), after the drug fully metabolized, mice were retested according to the same method.

For the experiment of LP glutamate receptors blockade, 2-amino-3-(3-hydroxy-5 -methyl-isoxazol-4-yl) propanoate (AMPA) and *N*-methyl-D-aspartate (NMDA) receptor antagonists, consisting of 10 mM of 2,3–Dioxo–6–nitro-1,2,3,4–tetrahydrobenzo [*F*] quinoxaline – 7-sulfonamide (NBQX) and 30 mM of

D-(-)-2-amino-5-phosphonopentanoate (AP5) respectively (Tocris, Bristol, UK), were dissolved in 0.9 % saline. 0.3 μl mixture or saline only was infused into the LP before behavior tests.

Analysis. Videos of whole behavior test were recorded by one or two video cameras and the offline analyses were performed by video tracking software (ANY-maze; Stoelting Co, Illinois, USA) and home customized matlab scripts. The video tracking software outputted several results frame by frame including: the animal positions (X, Y), staying zone divided by the users (side or center in testing arena), freezing score (*FS*), freezing state (freeze or not decided by the *FS* values) and animal speed (m/s).

The *FS* value is provided by ANY-maze software. This value is calculated from the difference of subject's shapes in two continuous frames, thus a more small *FS* value represents the less active of the subject. Animal keeps a totally immobile state can result that *FS* values almost equals to zero, and a experiential method for estimating a freezing state is *FS* values less than 30 lasting at least 300 ms (A stop freezing has to reach *FS* values more than 40 lasting at least 300 ms).

To describe the open space avoidance of mice in post-*UF* period, an additional parameter that the distance to center of testing arena was calculated: the center of arena was set as the zero point, the deviation of animal positions to the zero point was taken and normalized between the closest point (0) to the corner of the arena (1).

Perfusion and immunohistochemistry. ***Tissue section preparation and confocal imaging***. Mice were anaesthetized with chloral hydrate (10 % W/V, 300 mg/kg body weight, i.*P*.) and perfused transcardially with PBS, followed by ice-cold 4 % paraformaldehyde (PFA, 158127 MSDS, sigma) dissolved in PBS. Brains were removed carefully and post-fixed in PBS containing 4 % PFA at 4℃ overnight, and then equilibrated in PBS containing 25 % sucrose at 4℃ respectively until the brains sank to the *bottom* of the container. The coronal brains sections (40 µm) were taken using the cryostat microtome. Freely floating sections were washed with PBS, following which non-specific binding was blocked with 0.3 % TritonX-100 and 1 0 % normal goat serum (NGS, Jackson) in PBS for 1 h at room temperature. Sections were then incubated in the primary antiserum overnight which diluted in PBS with 3% NGS, 0.1% TritonX-100. Antibodies used were as follows: rabbit monoclonal anti-c-fos (dilution 1:200, 2250; Cell Signaling); mouse monoclonal anti-vesicular glutamate transporter, VGLUT2, (dilution 1:200, MAB5504; Millipore); mouse monoclonal anti-parvalbumin, (dilution 1:1000, MAB1572; Millipore); mouse monoclonal anti-GAD67, (dilution 1:100, MAB5406; Millipore) and Rabbit anti-CamKII, (dilution 1:250, AB52476; Abcam). After thoroughly washing with PBS, the sections were then incubated in the secondary antiserum Alexa fluor 488 or 594 goat anti-mouse IgG and Alexa fluor 488 or 594 goat anti-rabbit (all dilution 1:100, Jackson) with room temperature for 1 hours. After washing with PBS, the sections were mounted onto microscope slides and coverslipped with anti-fade reagent with DAPI or signal enhancer (Image-iT FX Signal Enhancer, Invitrogen).The

sections were then photographed and analyzed using Leica TCS SP5 laser scanning confocal microscope and ImageJ software.

Cell quantification. To determine the specificity of ChR2-mcherry expression in the SC, 6 coronal slices from the unilateral SC (*AP* from -3.50 mm to -4.30 mm, spaced 120 μm from each other) per mouse (n = 3) were included. Transduction efficiency was quantified using a confocal microscope (63× objective *N*.A.=1.4) by comparing the ChR2-mcherry cells with CamkIIa immunoreactive cells. Cell counting was performed manually using ImageJ. To quantification of expression of c-fos positive cells, 6 coronal slices from the bilateral SC (*AP* from -3.50 mm to -4.30 mm, spaced 300 μm from each other) per mouse (n=3, which were randomly selected from their group), or 6 coronal slices from the lateral amygdala (*AP* from -0.94mm to -1.94mm, spaced 120 μm from each other) per mouse (n=3) were included. The mice were perfused 30~60 minutes after the experiments and immunofluorescence staining were performed as previously described. Confocal images (20× objective NA 0.7) were acquired using identical pinhole, gain, laser settings. Cell counting was performed automatically using image pro plus (IPP) software.

Trans-synaptic tracer labeling. ***Plasmids***. For rabies virus–mediated trans-neuronal tracing, several AAV backbone plasmids was constructed in our lab or ordered from addgene. The plasmid AAV-EF1a-FLEX-GT was acquired from E.d. Callaway’s lab (26198; Addgene); the plasmid AAV-EF1a-DiO-RV-G was constructed by sub-cloning the coding region of Rv-G from AAV-EF1a-FLEX-GTB (26197;

Addgene) into the Dio cassette of the plasmid pAAV-EF1a-double floxed-hChR2(H134R)-EYFP (20298, addgene);. The plasmid AAV-CAG-GFP-ires-CRE was a gift from Neuronbiotech. All rAAV viruses were packaged into 2/9 serotype with titer at approximately $2x10^{12}$ genome copies per milliliter (Neuronbiotech).

Virus. The trans-multiple synaptic tracers, PRV-152, was belong to the recombinant pseudo rabies virus and herpes simplex virus respectively. The PRV-152 (kindly gift from L. W. Enquist), which expresses EGFP driven by CMV promoter, is a retrograde trans-multiple synaptic tracer and was prepared as previously described [6]. Briefly, PRV-152 was propagated in BHK21 cell line. Two days post infection, the cells were freeze-thaw two cycles. The culture solution was harvested and concentrated at 4000 g, 10 min. The supernatant were collected, filtered with 0.45 um filters (Millipore) and further concentrated into 100-fold through high speed concentration (Beckman Coulter), and tittered and stored in aliquot at -80 °C.

The trans-monosynaptic tracers-rabies virus (RV) and three cell lines for rabies propagation and tittering were kindly supplied by Callaway, E. M and prepared in our laboratory as previously described [7, 8, 9, 10]. Briefly, RV-G pseudo typed SAD19-ΔG -mcherry were propagated in B7GG cell, and harvested the supernatant with a titer of 105 infect unit/*ML*. To produce the EnvA-pseudotyped rabies, a Bhk-Enva cell was infected with filtered (0.45 um, Millipore) RV-G-SAD19-ΔG-mcherry (EnvA-RV-mcherry). Six hours post infection, the Bhk-Enva cell were digested with

0.25％ trypsin（Hyclone）to eliminate the contamination of RV-G pseudo typed rabies. During harvest of EnvA-RV-mcherry, the filtered supernatant was 2000~3000-fold concentrated through two cycles of high speed concentration as previous described. The concentrated aliquots were tittered in 293t-tva800 cell line. The final titer of EnvA-RV-mcherry was $2x10^8$ infecting unit per milliliter. All aliquots were stored at -80 °C.

Anatomy. All procedures were approved by Animal Care and Use Committees at the Wuhan Institute of Physics and Mathematics (WIPM), CAS. Adult male C57BL/6 (20-25 g) mice were used for virus tracing and all procedures on animals were performed in a BSL II animal facilities. Animals were anesthetized with chloral hydrate (400mg /Kg), and then placed in a stereotaxic apparatus (RWD, 68030, 68025). During surgery and virus injection, animals were kept anesthetized with isoflurane (1%). The skull above the targeted areas was thinned with a dental drill (STRONG, 90+102, Guangdong, China) and removed carefully. Injections were conducted with a 10 μl syringe (Hamilton, Nevada, USA) connected to a glass micropipette with 10~15 μm diameter tip.

To identify the relay station between the SC and LA, we combine the anterograde AAV labeling from the SC with rabies mediated retrograde and trans-monosynaptic tracing from the LA (*n* = 3 animals). The rAAV-CamkIIa-EYFP 2/9 was injected into SC, simultaneously the mixed helper AAV viruses containing AAV-CAG-GFP-ires-CRE ， AAV-EF1a-FLEX-GT and AAV-EF1a-DiO-RV-G,

(volume ratio: 1:2:3, total volume of 500 nl) were injected into the LA region with the following coordinate: (*AP*: -1.50 mm; *ML*:3.25 mm; *DV*:-4.00 mm). Two weeks later, the EnvA-RV-mCherry was injected into LA. Mice were sacrificed one-week after rabies infection.

Here, we chose the AAV-mediated trans-synaptic tracing using an EnvA-RV virus because the system has following advantages: Firstly, the starter cells can be restricted to the LA, shown by co-labeling with GFP from AAV and mCherry from Rabies. This ensure that RV labeled the LP neurons are raised from the LA rather than adjacent regions; Secondly, the relationship between the LA and the LP can be defined as mono-synaptic connection, excluding the possibility of fibers from the LP just passing through the LA; Lastly, more comprehensive labeling of the connections can be achieved. In this system, the signals in the starting neurons in the LA are amplified by AAV replication first, then the retrograde tracing is carried out by RV, overcoming the dosage effects with replication incompetent RV-dg-mCherry only (Although higher dosage can be used, but the spreading makes the starting site beyond the LA region).

To retrograde trace the afferent neural circuit from the LA, PRV-152 stocks was mixed with CTB alexa 594 (Molecular Probes, 0.05% in dH20) and calibrated in $1x10^9$ infecting unit per milliliter with 0.9 % saline. A volume of 300 nl of the mixed solution was stereotaxic microinjected into the LA region. Because PRV can replicate and trans-multiple synapses in a time-dependent manner, mice were sacrificed at

various stages of infection（36 h, 48 h and 60 h, respectively. *n* = 3 animals for each infection stage）.

For lesion studies, ibotenic acid (1% in dH2O, 300 nl) was injected ipsilaterally into the LP using similar method as with PRV/CTb injections. Ibotenic acid was injected 9 days before the injection of PRV/CTb in the LA (*n* = 3, mice were sacrified at 65h post-infection). As for the control test, an equal volume of 0.9 % saline was injected ipsilaterally into LP (*n* = 4, mice were sacrificed at 60h (*n* = 1) or 65h (*n* = 3) post-infection). The stereotaxic coordinate of LP is: (*AP*: -2.50mm; *ML*:1.50 mm; *DV*:-2.40 mm) .

Tissue section preparation and confocal imaging. All mice were deeply anaesthetized with an overdose of urethane (1g/kg) and were transcardially perfused with 400 *ML* of 0.9 % saline followed by 300 *ML* of 4 % paraformaldehyde (PFA) solution. The brain was peeled out, post-fixed in PFA overnight and dehydrated in 30% (W/V) sucrose solution for 3 days. The brain was sectioned coronally with Leica freezing microtome. Sequential brain sections, with a thickness of 40μm, were collected in 24 well plates (each 4 serial sections per well), and were stored at -20 °C. For immunohistochemistry, free-floating sections were washed in 0.1 M phosphate buffered saline (PBS) solution for 3 x 5 min, followed by an incubation in 10 % normal goat serum-PBS solution for 1-1.5 h. Sections were then incubated overnight in a rabbit polyclonal to GFP (dilution 1:1000, AB290; Abcam, for PRV 152

labeling). Sections were then washed in 0.1M PBS solution for 3 x 5 min and incubated in an FITC anti- rabbit antibody (dilution 1:200) for 1 h.

For florescent imaging of the brain section labeled by rabies or immunohistochemistry, the sections were washed with PBS, and wet mounted directly on Vecta-Shield mounting medium containing DAPI, sealed with nail polish, imaged with an inverted fluorescence confocal microscopy (Nikon).

Cell quantification. Whole brain sections were scanned sequentially under 10x objective of fluorescence confocal microscopy (Nikon) to find the retrograde RV:LA labeled cells. To quantification of PRV-152 positive cells expression in the SC, 4 coronal slices from the SC (*AP* from approximately -3.08 mm to -4.48 mm, spaced 320 μm from each other) per mouse were included (LP intact group: 60h (n = 1) or 65h (n = 2); LP lesioned group 65h (n = 3)). Confocal images (20× objective) were acquired and cell counting was automatic performed using image pro plus (IPP).

Statistical Analyses. One–way or two-way analysis of variance (ANOVA), Kruskal-Wallis one-way ANOVA (an extension of rank test to 3 or more groups), student’s t-tests, Mann–Whitney U-tests were used to determine statistical differences using SigmaPlot (version 12.0). Post hoc analysis was applied to test individual differences between subgroups. Detailed statistical analyses are provided in supplementary information. Statistical significance was set at *** $P < 0.001$; ** P <

0.01; * $P < 0.05$. All data values are presented as mean ± s.d. Graphs were made using SigmaPlot or Matlab.

Supplementary References

1. Franklin KBJ, Paxinos G. *The Mouse Brain in Stereotaxic Coordinates*. Elsevier/Academic Press (2008).

2. Witten IB, *et al.* Recombinase-Driver Rat Lines: Tools, Techniques, and Optogenetic Application to Dopamine-Mediated Reinforcement. *Neuron* **72**, 721-733 (2011).

3. Schmitzer-Torbert N, Jackson J, Henze D, Harris K, Redish AD. Quantitative measures of cluster quality for use in extracellular recordings. *Neuroscience* **131**, 1-11 (2005).

4. Anikeeva P, *et al.* Optetrode: a multichannel readout for optogenetic control in freely moving mice. *Nature neuroscience* **15**, 163-170 (2012).

5. Yilmaz M, Meister M. Rapid innate defensive responses of mice to looming visual stimuli. *Current biology : CB* **23**, 2011-2015 (2013).

6. Smith BN, *et al.* Pseudorabies virus expressing enhanced green fluorescent protein: A tool for in vitro electrophysiological analysis of transsynaptically labeled neurons in identified central nervous system circuits. *Proceedings of the National Academy of Sciences of the United States of America* **97**, 9264-9269 (2000).

7. Wickersham IR, *et al.* Monosynaptic restriction of transsynaptic tracing from single, genetically targeted neurons. *Neuron* **53**, 639-647 (2007).

8. Osakada F, Mori T, Cetin AH, Marshel JH, Virgen B, Callaway EM. New Rabies Virus Variants for Monitoring and Manipulating Activity and Gene Expression in Defined Neural Circuits. *Neuron* **71**, 617-631 (2011).

9. Weible AP, *et al.* Transgenic Targeting of Recombinant Rabies Virus Reveals Monosynaptic Connectivity of Specific Neurons. *Journal of Neuroscience* **30**, 16509-16513 (2010).

10. Wall NR, Wickersham IR, Cetin A, De La Parra M, Callaway EM. Monosynaptic circuit tracing in vivo through Cre-dependent targeting and complementation of modified rabies virus. *Proceedings of the National Academy of Sciences of the United States of America* **107**, 21848-21853 (2010).
